# Predicting clinical outcomes in *Helicobacter pylori-*positive patients using supervised learning through the integration of demographic and genomic features

**DOI:** 10.1186/s12876-025-04595-3

**Published:** 2026-01-29

**Authors:** Venkatesh Narasimhan, Sreya Pulakkat Warrier, Jobin Jacob John, Monisha Priya T., Niriksha Varadaraj, Greeshma Grace Thomas, Balaji Veeraraghavan

**Affiliations:** 1 Department of Clinical Microbiology, Christian Medical College and Hospital, Vellore, India; 2 Department of General Medicine, Christian Medical College and Hospital, Vellore, India

**Keywords:** Helicobacter pylori, Gastric cancer, Supervised machine learning, Genomic sequence-based feature extraction, Ensemble learning, SHAP interpretability, Machine learning

## Abstract

**Background:**

*Helicobacter pylori (H. pylori)* infection is widespread globally and is linked to outcomes ranging from chronic gastritis to gastric cancer. However, only a minority of infected individuals progress to malignancy, influenced by a mix of bacterial, host, and environmental factors. Current predictive approaches are limited due to relying mainly on clinical and lifestyle data. Genomic approaches have been sparsely used, and thus their incorporation into machine learning models could ensure early and personalized detection. This study aimed to evaluate the impact of integrating host metadata with genomic features from *H. pylori* to predict gastric cancer outcomes and identify associated variables.

**Methods:**

One thousand three hundred sixty-three publicly available *H. pylori* genomes with associated host information between 1991 and 2024 were collected from NCBI and EnteroBase. Demographic features, virulence genes, sequence-derived and variant-based features were extracted. Machine learning models were then developed to classify infection outcomes into gastric cancer and non-gastric cancer and trained using internal cross-validation folds within the training set comprising 80% of the dataset. Logistic regression, an interpretable baseline model, was compared against higher-performance ensemble models (XGBoost, Random Forest). Final model performance was assessed on the held-out test set using recall, precision, AUROC, and AUPRC curves.

**Results:**

The logistic regression model achieved a recall of 0.737 (95% CI: 0.637–0.830) for gastric cancer and an AUROC of 0.830 (95% CI: 0.779–0.880). Both XGBoost and Random Forest models outperformed the baseline model with AUROC values ranging from 0.950 to 0.954 (95% CI: 0.904–0.976). Black-box model recall for gastric cancer detection improved compared to the baseline by 8.14% for XGBoost (0.797, 95% CI: 0.711–0.877), and 11.3% for Random Forest (0.820, 95% CI: 0.734–0.896). Across models, patient age consistently emerged as the strongest predictor of gastric cancer, with several sequence-derived genomic features beyond pre-established virulence genes contributing to the infection outcome differences.

**Conclusion:**

This study demonstrates that combining pathogen genomics with host demographics uncovers novel risk factors and ensures early detection with high predictive power. The use of explainability methods like SHAP allows for greater interpretability by clinical professionals and improves informed decision-making processes. While internal validation showed strong performance, external validation on independent data and translation into clinical practice is necessary using broader, diverse datasets, along with the inclusion of additional host and lifestyle variables.

**Supplementary Information:**

The online version contains supplementary material available at 10.1186/s12876-025-04595-3.

## Background

*Helicobacter pylori* (*H. pylori*) is a highly motile, helical and gram-negative bacterium that is known for colonising the human gastrointestinal tract, and primarily associated with chronic active gastritis. *H. pylori* infection is strongly linked to the development of serious gastrointestinal diseases and accounts for up to 90% of duodenal ulcers, and around 80% of stomach ulcers and 90% of gastric carcinoma and mucosa-associated lymphoid tissue (MALT) lymphoma cases [1]. In accordance with the burden posed by these diseases, the World Health Organisation (WHO) recommended prioritising *H. pylori* eradication strategies in 2014 to help in reducing stomach cancer mortality rates worldwide. At the global level, almost half of the world’s population is infected with *H. pylori*, with more than 50% of the infected people living in resource-limited regions, which are often characterized by suboptimal sanitation and healthcare access, and around 34% belonging to industrialized countries [2, 3]. Gastric cancer is also relatively uncommon among younger individuals with fewer than 10% of cases occurring before the age of 45. There is a sharp spike in the lifetime risk of gastric cancer for infected individuals, with the probability being nearly double compared to uninfected individuals [4]. The bacterium’s ability to thrive in the acidic gastric environment has been linked to specialized virulence mechanisms. These help it facilitate persistent colonization and chronic inflammation, which dictate the host’s gastritis phenotype and disease trajectory. There are several *H. pylori* genes that have been implicated in the development of gastric cancer, although none of them have been discovered using machine learning and there have been no studies, to the best of our knowledge, that have used pathogen-only genomic factors for gastric cancer risk prediction. The cytotoxin-associated gene A (*cagA)* gene, encoding the CagA protein is found at the end of the cag pathogenicity island (cagPAI), and is associated with increased cancer risk and severe infections [5]. The marker is more discriminative in Western countries on account of only around 60% of strains carrying *cagA*, compared to over 90% of East Asian strains being *cagA*-positive [5]. The study by Kong et al. (2025) found gastric cancer-derived strains to consistently carry the *cagA* gene with gastritis strains often lacking it [6]. The vacuolating cytotoxin A (*vacA)* gene encodes the vacA cytotoxin which helps in inducing vacuole formation, autophagy modulation and evading immune responses [7, 8]. Strains harbouring the *vacA* s1/m1/*cagA* + genotype show a 4.8-fold increased risk of progressing to precancerous lesions compared to *vacA* s2/m2/*cagA*- strains [9]. The *babA2* gene, encoding the blood group antigen binding adhesin (BabA) that binds to mucosal Lewis^b^ blood group antigens, helps in aiding colonisation and increasing bacterial load [10]. The triple positive genotype possessing *babA2*,* vacA* s1 and *cagA* genes offer stronger gastric cancer discrimination than *vacA* and *cagA* alone [11]. The outer inflammatory protein A (*oipA*) gene promotes epithelial attachment, IL-8-mediated inflammation and disruption of cell turnover, and is often found co-expressed with other virulence genes [12–17]. *homB* gene has been found to aid adhesion and IL-8 induction, while *sabA* promotes neutrophil activation and inflammation through binding to sialylated Lewis antigens (sLe^X^) [18–21].

The *htrA* gene is closely linked to tumor suppression and metastasis, and the *iceA1* gene has also been significantly associated with gastric cancer [22–24]. The *hopQ* type I allele is linked with increased gastric cancer and gastric ulcer risk, especially in East Asia where it is commonly present with *cagA*, while type II *hopQ* is more frequent in Western strains [25].

Diagnosing *H. pylori* infection is reliant on a combination of methods. These include invasive methods such as endoscopy and biopsy, and non-invasive approaches like urea breath tests, stool antigen tests, and serological assays. The diagnostic tool choice is guided by a lot of factors like patient history, clinical presentation and resource availability. Typical eradication therapy usually involves a potent acid suppressant with antibiotics and bismuth compounds in some cases. Curbing the alarming rise of antibiotic resistance among *H. pylori* strains relies on judicious antibiotic use and resistance surveillance methods [26].

Understanding the genetic diversity of *H. pylori* and its associated phenotypic variability can be used to build accurate prediction models using machine learning approaches. This could help improve therapeutic approaches by stratifying patients based on infection outcomes [27].

Traditional statistical methods struggle to capture the complex, non-linear interactions that influence disease outcomes using a mix of demographic and genomic variables. High dimensional and heterogeneous datasets cannot be adequately modelled using linear statistical techniques. Machine learning is capable of modeling complex relationships without presupposing linearity and can improve predictive performance. To preserve interpretability, logistic regression was used as a baseline white-box model, while XGBoost and Random Forest were utilized due to their ability to handle multicollinearity, heterogeneous feature types and non-linear interactions.

**Fig. 1:**
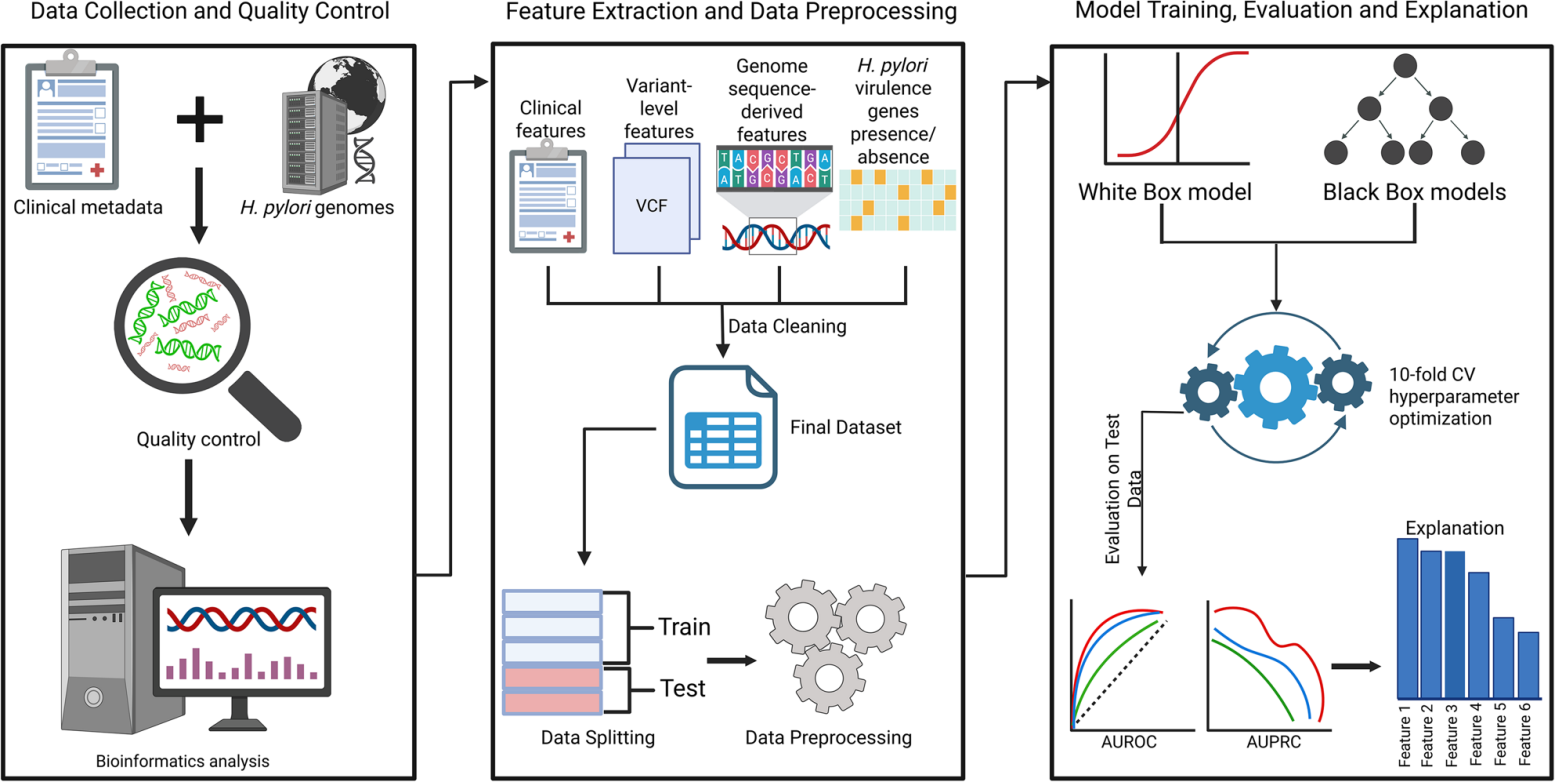
Overview of the machine learning workflow for classification of gastric cancer and non-gastric cancer using *H. pylori* genomes and clinical data The workflow consists of three stages, depicted from left to right, with arrows indicating the flow of the stages: (1) Data collection and quality control, host clinical metadata and *H. pylori* whole genomes are integrated and filtered for quality, and bioinformatics analysis (variant calling); (2) Feature extraction and data preprocessing (normalization, one-hot encoding, label encoding), including clinical features, variant-level features from VCF files, and genome sequence-derived features and presence/absence data of *H. pylori* virulence genes to construct the final dataset, followed by data preprocessing; and (3) Model training, evaluation and explanation, involving binary classification using white-box (logistic regression) and black-box models (XGBoost and Random Forest) with 10-split cross-validation and hyperparameter bayesian optimization, performance evaluation using AUROC and AUPRC metrics, and finally feature importance interpretation. Icons sourced from BioRender (https://app.biorender.com/)

The clinical outcomes of *H. pylori* infection considerably vary across geographic populations and are influenced by a variety of factors like bacterial genotype diversity, host immune responses, and environmental factors [28]. Despite there being some studies on predicting gastric cancer in *H. pylori* infected patients, they have primarily relied on clinical and lifestyle data and often included patients without confirmed *H. pylori* infection [29–32]. An XGBoost model to predict future gastric cancer by Taninaga et al. (2019) [29] utilized routine check-up data including age, sex, *H. pylori* infection status, endoscopy and lab values and achieved an AUROC of 0.899, while the XGBoost models built by Jiang et al. (2022) [30] and Afrash et al. (2023) [31] used lifestyle factors like food intake, smoking, history of gastric cancer in first-degree relatives to achieve AUROC values of 0.896 and 0.849 respectively. A logistic regression model built using electronic health record-derived factors like age, sex, race, smoking, and anemia by Kim et al. (2024) reported that older age (OR ≈ 1.16), male sex (OR ≈ 1.98), Black/Asian race (OR ≈ 4–5), smoking (OR ≈ 1.77) and pernicious anemia (OR ≈ 6.12) all resulted in higher non-cardia gastric cancer risk [33]. The studies that have taken advantage of *H. pylori* pathogen genomics to build machine learning models are primarily antimicrobial resistance prediction studies [34, 35]. Since traditional clinical studies often ignore pathogen genomic diversity which play a heavy role in modulating disease risk, Haley et al. (2015) emphasize the need for integrating host genetics, environmental and demographic factors along with bacterial virulence properties to determine disease outcome [36]. To our knowledge, there have been no studies that have integrated clinical host metadata, aggregated variant annotation features from VCF files, virulence gene presence and absence data, and sequence-derived features from *H. pylori* genomes in a single, unified machine learning framework. In this study, we focused solely on *H. pylori* infected patients and developed a machine learning pipeline to classify gastric cancer versus non-gastric cancer cases using a combination of the above-mentioned features. A comprehensive summary of the study workflow is provided in Fig. 1. In the presence of possible confounders like age and geography, our integrative approach prioritizes predictive performance for clinical utility, and is an attempt to identify variables that warrant further investigation in mechanistic studies, and not to establish causation. We aim to provide researchers with novel genomic markers that can be further analyzed for causation in wider studies, along with the validation of established causative factors of gastric cancer like age. We hope this will improve stratification of infection responses and prevent delayed care for high-risk patients, along with increasing physician trust and augmenting decision-making.

## Methods

### Data collection

*H. pylori* genomes from NCBI and EnteroBase were filtered for the presence of host-age, host-sex, host geographical location and disease phenotype information, resulting in 1,587 genomes. The genomes from EnteroBase were pre-assembled and case controls were excluded.

NCBI Biosample Metadata was similarly downloaded and case controls and duplicates were excluded. Pre-assembled genomes from GenBank and RefSeq were downloaded using NCBI Datasets, while Sequence Read Archive (SRA) database sequences consisting of high-throughput short reads were downloaded through Fastq-dump from SRA Toolkit (version 3.2.1) .

### Genome quality control and assembly

The unassembled short reads were assembled with the help of SKESA (version 2.4.0). Quality control was carried out using Pathogenwatch (version 23.2.1) to filter out contaminated genomes belonging to the wrong species, resulting in 781 EnteroBase downloaded genomes and 582 genomes from NCBI. The final dataset consisted of 1,363 genomes which passed the quality control step and were collected in the years spanning from 1991 to 2024.

### Feature extraction

In order to develop a machine learning model well-equipped to predict gastric cancer status in *H. pylori* infected patients, a diverse set of features was included in the final dataset. These included clinical metadata from NCBI and EnteroBase, gene presence and absence information obtained with the help of BLASTN-based homology searches, aggregated variant annotation features from VCF files and genome-derived sequence descriptors.

### Clinical and epidemiological features

The clinical metadata associated with each *H.pylori* genome was collected from EnteroBase and NCBI databases (Supplementary Table S1). The age, sex, and geographical location of the host along with their disease phenotype was extracted. The continent-wise split of the dataset is as follows: 870 samples from Europe, 282 samples from North America, 115 samples from South America, 95 samples from Asia. With the help of clinical expertise, this disease phenotype was converted into one of two labels: gastric cancer (including cases of atrophic gastritis and metaplasia) and non-gastric cancer (including cases of ulcers and non-atrophic gastritis). This served as the label for the machine learning model and was appropriately encoded as mentioned in later sections.

### Gene presence and absence features

In addition to the clinical metadata, gene variant presence and absence information was also derived by identifying relevant gene variants associated with gastric and non-gastric cancer phenotype through exhaustive literature review.

These reference gene variant sequences linked to certain strains were extracted from a collection of genomes. These included *cagA*, *babB*, *oipA*, *sabA* and *vacA *s1m1 from *H.pylori* strain 60190; *babA2* from *H.pylori* strain ATCC 43504; *hopQI* from *H.pylori* strain J99; *homB*, *hrgA*, *htrA*, *iceA1* from *H.pylori* strain 26695; *hopQII* from Tx30a.

Each of these assembled genomes were formatted into a separate BLAST compatible database. Each gene sequence was queried against each of the 1,363 genome databases with the help of BLASTN (version 2.12.0). A gene was considered present in a genome if the top hit satisfied the below criteria:

- Percentage identity > = 90%.
- Query coverage > = 80%.
- E-value < = 1e-5.

The presence of each gene was encoded as 1 and the absence as 0, resulting in a binary feature set (Supplementary Table S2). These thresholds were selected in order to balance sensitivity and specificity and to ensure accurate homologous gene detection by minimizing false positives.

### Assembled genomic sequence derived features

To capture additional biological information, numerical sequence descriptors were extracted directly from the genomes using two Python-based packages, iFeatureOmega (version 1.0.2) and MathFeature (version 1.0) (Table 1).

**Table 1:** Feature types utilised in this study and the respective package

Package	Feature type	# Features
iFeatureOmega	Z-curve (144-bit)	144
	Kmer Type 1	64
	RCKmer Type 1	32
	Nucleic Acid Composition (NAC)	4
	Multivariate Mutual Information (MMI)	30
	CKSNAP type 1	64
MathFeature	Shannon Entropy	3
	ORF-Based Features	10

These sequence-based features were computed by the packages at a contig level, which were then averaged in a length-weighted manner to generate a single representative feature vector per genome (Supplementary Table S3).

Specifically, for a given feature $\:f$, the genome-level feature average $\:F$ was calculated as:

1 $$\:F=\frac{{\sum\:}_{i=1}^{n}{f}_{i}\times\:\:{l}_{i}}{{\sum\:}_{i=1}^{n}{l}_{i}}\:$$

where:

- $\:{f}_{i}$ is the feature value for the $\:i$-th contig,
- $\:{l}_{i}$ is the length (in base pairs) of the $\:i$-th contig,
- and $\:n$ is the total number of contigs for that genome.

This method ensured that longer contigs which typically provide more reliable sequence information would contribute proportionally to the final feature representation.

### Variant-Based features from VCF files

Variant level features were extracted from genome-wide mutation profiles to include impact about sequence variation and their functional effects. The strain 26695 was used as a reference to call variants using Snippy (version 4.6.0), a rapid bacterial variant calling pipeline, on the assembled *H.pylori* genomes. Following variant calling, the Snippy-created VCF files were passed to another tool known as bcftools (version 1.2.1) for identifying the number of SNPs and MNPs. Using bcftools’ (version 1.2.1) stats module, the raw counts of the following features were extracted: SNPs, MNPs, Indels, and all possible nucleotide changes (e.g.,A → T, T → C, etc.). The variant effect functional annotations were also extracted from the VCF files to classify sequence changes based on their predicted effects on coding regions. The total counts of each variant effect were calculated per genome. These included types of mutations such as missense variants, frameshift mutations, mutations that affect start codons and combinations of these. For each genome, the number of variants falling into each functional category was counted and recorded as individual features.

These metrics were aggregated into per-genome features to help capture unexplored signals associated with the gastric cancer phenotype (Supplementary Table S4).

### Data preprocessing

A comprehensive series of steps were carried out to preprocess the dataset to make it amenable for model training (Supplementary Table S5). The versions of scikit-learn, Pandas, matplotlib and Python used were 1.6.1, 2.2.3, 3.10.1 and 3.12.3.

Minor corrections were carried out to standardise categorical entries including correcting typographical errors, standardising the case of the characters and removing entries where the host sex was indeterminate, resulting in 1,362 genomes.

The dataset was then split into training sets and test sets using the StratifiedShuffleSplit module from scikit-learn (version 1.6.1) (80% training set, 20% test set) and was stratified along the labels to maintain class distribution in both the training and test data. For the input features, preprocessing for the input features involved normalisation using the MinMaxScaler module and one-hot encoding using the OneHotEncoder module in scikit-learn.

Label encoding was used to convert the categorical class labels of ‘Gastric cancer’ and ‘Non-gastric cancer’ into numeric representations of 0 and 1 respectively using the LabelEncoder module in scikit-learn.The same processes of normalisation and encoding were also applied separately to the test set to ensure that the preprocessing steps were consistent across training and test datasets.Normalization and encoding were fit only on the training set; the transformations performed on the test set were done independently to prevent any form of information leakage. The resulting dataset was rendered suitable for further feature selection and model training steps.

### Batch effect visualization, subgroup performance and sensitivity analyses

Batch effect visualization, subgroup and sensitivity analyses were performed to evaluate the model’s reliability, its generalizability and performance boundaries across patient populations and data sources.

#### Principal component analysis (PCA) for batch effect visualization

PCA was conducted on the complete encoded feature set (*n* = 1,362 samples) to visualize any potential clustering, or separation by data source. Extraction of the first three principal components was followed by plotting the samples in PC1-PC2 space colored by source. To quantitatively assess source-based clustering, the silhouette score was calculated. This typically ranges from − 1 (incorrect clustering) to + 1 (well-separated clusters), with values near 0 indicating overlapping distributions. A low silhouette score would be indicative of minimal batch effects, as samples from different sources would be intermixed instead of forming distinct technical clusters.

#### Geographic stratification analysis

In order to assess potential geographic bias and evaluate model performance across different populations, performance metrics were calculated separately for European (*n* = 169), North American (*n* = 61), South American (*n* = 25), and Asian (*n* = 18) cohorts within the test set. For each geographic region, the AUROC, AUPRC, precision, recall, specificity, and F1-score values were calculated. This was done to identify any potential performance heterogeneity across populations reflecting differences in *H. pylori* strain diversity, host genetics, or environmental factors.

#### Age-stratified calibration analysis

To assess whether model calibration varied across patient demographics and to identify any potential systematic miscalibration in specific age groups, age-stratified calibration analysis was performed. Patients were divided into three clinically relevant age groups based on gastric cancer epidemiology: <45 years (low baseline risk), 45–65 years (intermediate to high risk), and ≥ 65 years (highest risk). For each age stratum, calibration curves were generated to show the relationship between mean predicted probabilities (binned into 10 equal-width bins) and observed outcome frequencies. Calibration quality was quantified using Brier scores and Expected Calibration Error (ECE) as defined in Eqs. (2) and (3). This stratified analysis aimed to identify any resulting age-specific performance boundaries, and also to assess if predicted probabilities were systematically over- or under-confident in specific patient demographics.

#### Source-stratified performance evaluation

To evaluate any potential batch effects arising from data heterogeneity across sources (NCBI vs. EnteroBase), model performance was evaluated separately on NCBI-derived samples (*n* = 121) and EnteroBase-derived samples (*n* = 152) within the held-out test set. This analysis aimed to assess whether predictive performance was consistent across different data sources that could vary in sequencing platforms, collection years, or genomic assembly methods.

#### Leave-One-Source-Out (LOSO) cross-validation

In order to assess model generalizability across data sources, as well as evaluate robustness to potential batch effects, leave-one-source-out cross-validation was implemented on the complete dataset (*n* = 1,362). In each fold, the models were trained on all samples from one source (NCBI or EnteroBase) and tested on samples from the other source. This was done in an effort to mimic real-world scenarios, where models trained on data from one institution are typically also applied to data from differing sources. All three models (logistic regression, XGBoost, Random Forest) were trained using the same hyperparameters identified through Bayesian optimization and the same 93-feature subset selected via ShapRFECV. SMOTE-NC was applied within each training fold to handle class imbalance.

All these analyses aimed to collectively assess model performance boundaries across patient subgroups differing in geography and age, ascertain the reliability of probability estimates through calibration, as well as robustness to technical variation arising from different data sources.

### Feature type-specific analysis

While statistical methods exist for controlling confounding factors through regression in linear models, these approaches are not applicable to predictive ensemble models due to their non-linear assumptions. To evaluate the relative contribution of demographic versus genomic features to model performance, all three machine learning models were trained and evaluated on three distinct feature sets:

1. Demographic features only (age and geographic location).
2. Genomic features only (excluding all demographic variables).
3. Combined feature set (both demographic and genomic features).

This analysis aimed to assess whether genomic features provide predictive value beyond easily accessible demographic information, and whether demographic features (particularly age) dominate predictions at the expense of genomic signals. Models were trained using the same hyperparameters and cross-validation strategy as the full-feature models, with performance evaluated on the held-out test set using AUROC, AUPRC, and gastric cancer-specific recall metrics.

### Feature selection

Feature selection was performed on all the models to mitigate overfitting, enhance generalization and improve the model’s explainability. The ShapRFECV module in the probatus package (version 3.1.2) was used to select features. SHAP-based feature selection was performed solely on the training set by selecting features with the highest mean SHAP score across the 10-split stratified shuffle cross-validation splits used internally to evaluate model performance. The features with the lowest SHAP scores were iteratively removed (step = 0.2, removing 20% of features per iteration) to get the final optimal feature set of 93 features which maximized recall performance. The models were trained using the selected features while the test set (20% holdout) remained completely independent throughout the feature selection and model training processes.

### Model training and development

A logistic regression classifier was trained as the baseline model and chosen for its white-box functionality and for its ease of interpretability. A random state of 26 was used to maintain reproducibility.

Class imbalance in the dataset was handled using SMOTE-NC [37]. It was preferred over SMOTE due to its ability to handle both categorical and continuous features by generating synthetic samples for the minority class while retaining the categorical nature of categorical variables. It was applied within a generated pipeline to the training splits within each cross-validation split to ensure that synthetic samples did not influence the model evaluation step on the validation step.

The full model training pipeline was built using imblearn’s make_pipeline function and had SMOTE-NC and logistic regression classifier as its two components.

Cross-validation was carried out using StratifiedShuffleSplit by splitting the dataset into 10 splits for robust evaluation, with the stratification step preserving the class distribution during both model training and evaluation. Recall was selected as the scoring metric to prioritise accurate identification of gastric cancer cases and minimise any false negatives. After cross-validation, the model was refitted on the entire training dataset to optimise its performance before applying it to the test set.

An XGBoost classifier was trained as the black-box model and chosen for its black-box high performance. The steps taken for preprocessing the dataset were identical to the baseline model, including the chosen random state.

Categorical feature indices were identified and used with SMOTE-NC and a pipeline to first oversample and then train an XGBClassifier on the reduced feature set. Hyperparameter tuning was carried out using the BayesSearchCV function from scikit-optimize package, which employs Bayesian optimization to automatically search for the best hyperparameters to enhance the performance of the model.

Probability calibration was also performed on the XGBoost and Random Forest classifiers using CalibratedClassifierCV specifically using sigmoid regression to ensure that the predicted probabilities better reflected the true likelihood of outcomes. Brier score and Expected Calibration Error (ECE) were computed to assess the quality of calibration.

The Brier score quantifies the mean squared difference between predicted probabilities and actual outcomes:

2 $$\:Brier\:Score\:=\:\frac{1}{N}{\sum\:}_{i=1}^{N}({{{f}_{i}\:-\:{o}_{i}{)}^{2}}_{}}_{}$$

Where:

- *N* = total number of samples.
- *f*_*i*_ *=* predicted probability of sample *i*.
- *o*_*i*_ *=* observed outcome for sample *i* (0 = gastric cancer, 1 = non-gastric cancer).

The ECE measures the average difference between predicted confidence and actual accuracy across probability bins:

3 $$\:ECE\:=\:{\sum\:}_{m=1}^{M}\frac{\left|{B}_{m}\right|}{n}\left|acc\left({B}_{m}\right)-conf\left({B}_{m}\right)\right|$$

Where:

- *M* = number of bins
- *B*_*m*_ = samples in bin m
- *n* = number of samples
- *acc(B*_*m*_*)* = accuracy of predictions in bin m
- *conf(B*_*m*_*)* = average predicted probability in bin m

Lower values in both Brier score and ECE indicate better-calibrated predictions. Before-and-after calibration plots were generated to visualize the improvement in probability calibration, with both metrics displayed for comprehensive assessment of calibration quality.

After cross-validation, the model was refitted on the entire training dataset to optimise its performance before applying it to the test set. A Random Forest black-box classifier was trained and evaluated in the same manner as the XGBoost model. All the trained models were evaluated on the encoded test set using the trained pipeline to assess its generalization abilities on unseen data.

### Model evaluation

A classification report was generated in order to provide the values of key metrics like precision, recall and F1-score. These metrics were calculated for both the positive class (gastric cancer) and the negative class (non-gastric cancer), along with their 95% confidence intervals obtained using bootstrap resampling. Specificity was explicitly reported for the negative class, while sensitivity (recall) was reported for the positive class.

Along with this, the Area Under the Receiver Operating Characteristic curve (AUROC) and The Area Under the Precision-Recall curve (AUPRC) was plotted to detect a trade-off between correct identification of true positives and minimization of false positives and the proportion of true positives among all predicted positives against the proportion of actual positives identified by the model respectively. The Area Under the Curve (AUC) score and Average Precision (AP) score was calculated with 95% confidence interval. Additionally, a confusion matrix was plotted for both raw counts and percentages per cell, annotated with accuracy, sensitivity and specificity values. Calibration quality was quantified using Brier scores, with lower scores indicating that the model’s predicted probabilities were closer to the true outcome frequencies and therefore better calibrated.

### Post-hoc explanations

To interpret model predictions and understand the contribution of each feature, post-hoc explainability techniques were performed using SHAP [38, 39], which has been implemented as a Python package (version 0.47.1). The SHAP values were calculated after the normalization of the dataset; this ensures that the SHAP values reflect feature contributions enabling fair comparison across features with different ranges. The average impact of each feature on the model’s output was visualized using SHAP summary plots for the three models (logistic regression (baseline), XGBoost, and Random Forest). In addition to global explanations, local explanations were generated for specific test samples using SHAP waterfall plots. These plots decomposed an individual prediction into contributions from each feature relative to the model’s expected value, with the explained class chosen as either the model’s predicted class or a user-specified class index (0 for gastric cancer, 1 for non-gastric cancer). For each explained sample, the predicted class label and corresponding predicted probabilities for both classes were reported. These plots were used to illustrate the global and local feature importance across the whole dataset. These explainability techniques aid interpretation of models and help in assessing the consistency of feature contributions across different models.

## Results

Classification models were developed to group subjects into the gastric cancer and non-gastric cancer classes.

After the train-test split and before applying SMOTE-NC to handle class imbalance, there were 332 gastric cancer samples and 757 non-gastric cancer samples in the training set. After applying SMOTE-NC to oversample the minority class (gastric cancer), there were 757 samples of gastric cancer and 757 samples of non-gastric cancer in the training set. Prior to model training, feature selection was performed using ShapRFECV with cross validation to identify the most informative features for gastric cancer classification.

Cases of ulcers and non-atrophic gastritis were clubbed under the label ‘non-gastric cancer’ to keep with the model’s aim of determining the patient’s risk of gastric cancer, due to marked genomic differences in the virulence genes of gastric cancer-inducing *H. pylori* genomes compared to those causing ulcers and non-atrophic gastritis.

### Overall model performance

All the models were trained on the selected feature set containing both demographic and genomic features. The feature selection reduced the initial feature set from 533 to 93 features, with the optimal subset determined through 10-split cross validation based on recall scores (Supplementary Table S6). Hyperparameter optimization was conducted using bayesian optimization with 10-split cross validation for both the black-box models. For XGBoost, the optimal parameters were: n_estimators = 1000, max_depth = 7, colsample_bytree = 1.0, subsample = 0.601, and learning_rate = 0.0279. Random Forest achieved optimal performance with n_estimators = 955, max_depth = 10, and min_samples_split = 3. Complete hyperparameter configurations are provided in Supplementary Table S7.

#### Logistic regression (baseline model)

**Fig. 2:**
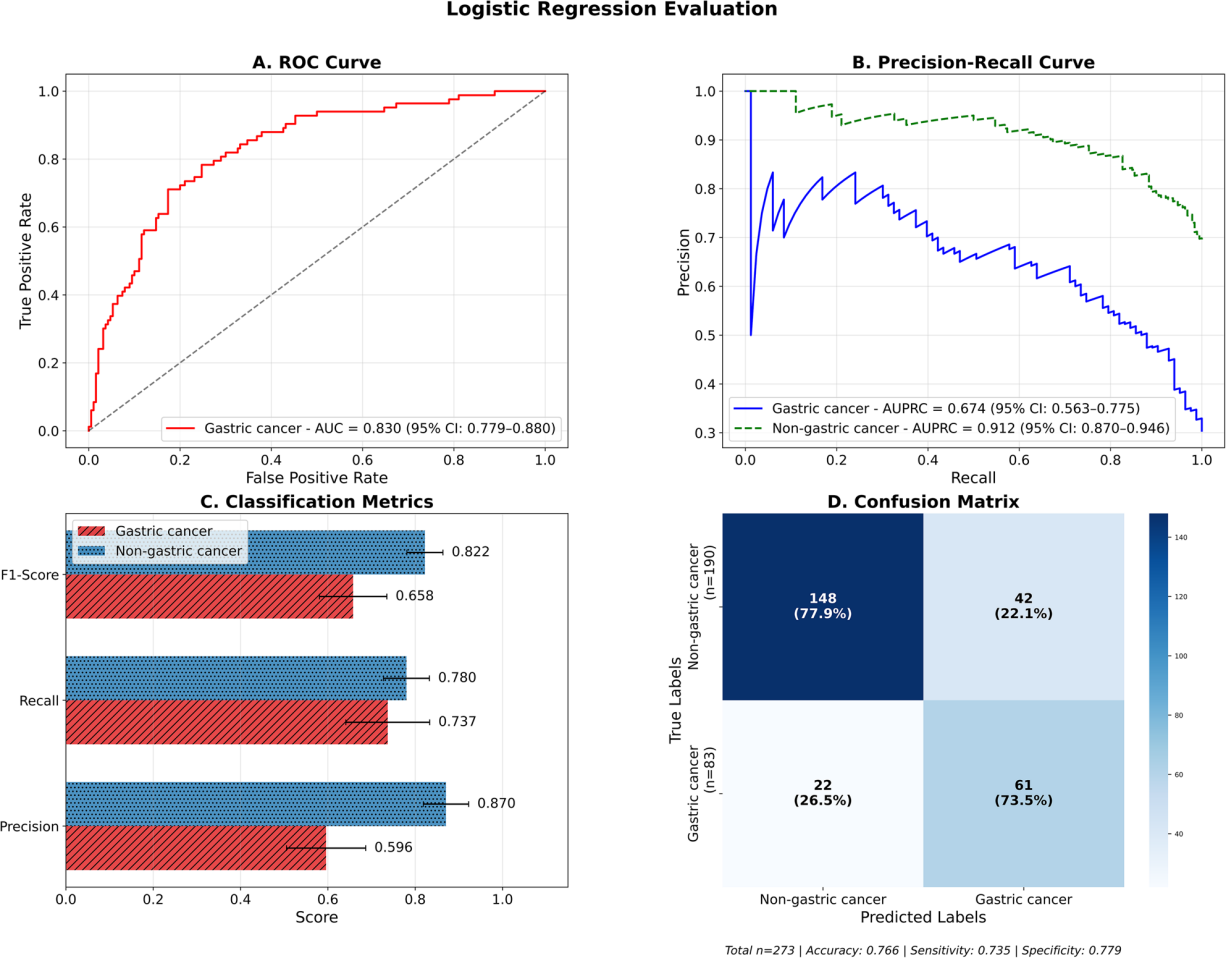
Logistic regression model performance for gastric cancer vs non-gastric cancer classification **A** ROC curve showing discriminative ability with AUC of 0.830 (95% CI: 0.779-0.880). **B** Precision-recall curves for both gastric cancer (AUPRC = 0.675, 95% CI: 0.563-0.775) and non-gastric cancer (AUPRC = 0.912, 95% CI: 0.870-0.946) classes. **C** Classification performance metrics comparing F1-score, recall, and precision between gastric and non-gastric cancer predictions. **D** Confusion matrix displaying true versus predicted labels with overall accuracy of 76.6%, sensitivity of 73.5%, and specificity of 77.9%

Logistic regression was used as a baseline classifier to evaluate initial predictive performance on the binary classification task of gastric cancer vs. non-gastric cancer. On applying oversampling using SMOTE-NC, the confusion matrix (Fig. 2D) revealed that the model correctly classified 61 out of 83 gastric cancer cases (73.5% sensitivity) and 148 out of 190 non-gastric cancer cases (77.9% specificity). The model produced 22 false negatives (26.5% of gastric cancer cases misclassified as non-gastric) and 42 false positives (22.1% of non-gastric cancer cases misclassified as gastric), resulting in an overall accuracy of 0.766 (95% CI: 0.718–0.813). The model displayed balanced performance across key metrics (Fig. 2C), with precision values of 0.596 (95% CI: 0.505–0.686) for gastric cancer and 0.870 (95% CI: 0.816–0.92) for non-gastric cancer classification.

The recall performance showed class-specific differences, with gastric cancer class recall of 0.737 (95% CI: 0.637–0.830), and non-gastric cancer recall of 0.780 (95% CI: 0.726–0.832). This resulted in F1-scores of 0.658 (95% CI: 0.580–0.734) and 0.822 (95% CI: 0.779–0.862) for gastric and non-gastric cancer classes respectively (Fig. 2C). The model also achieved a moderate AUPRC of 0.674 (95% CI: 0.563–0.775) for gastric cancer and 0.912 (95% CI: 0.870–0.946) for non-gastric cancer classes (Fig. 2B). The discriminatory power of the model denoted by an AUROC of 0.830 (95% CI: 0.779–0.880) was equal for both the classes. These findings are illustrated in Fig. 2A. Detailed performance metrics with CIs are provided in Supplementary Table S8 and the classification report of the model is provided in Supplementary Table S9. Overall, logistic regression proved to be a reasonable baseline with good performance. The lower recall for gastric cancer detection (73.6%) shows the necessity for more complex models that enhance the detection of the minority gastric-cancer class. Model calibration analysis showed reasonable probability confidence with a Brier score of 0.165 and ECE of 0.117 (Supplementary Figure S1).

#### XGBoost

**Fig. 3:**
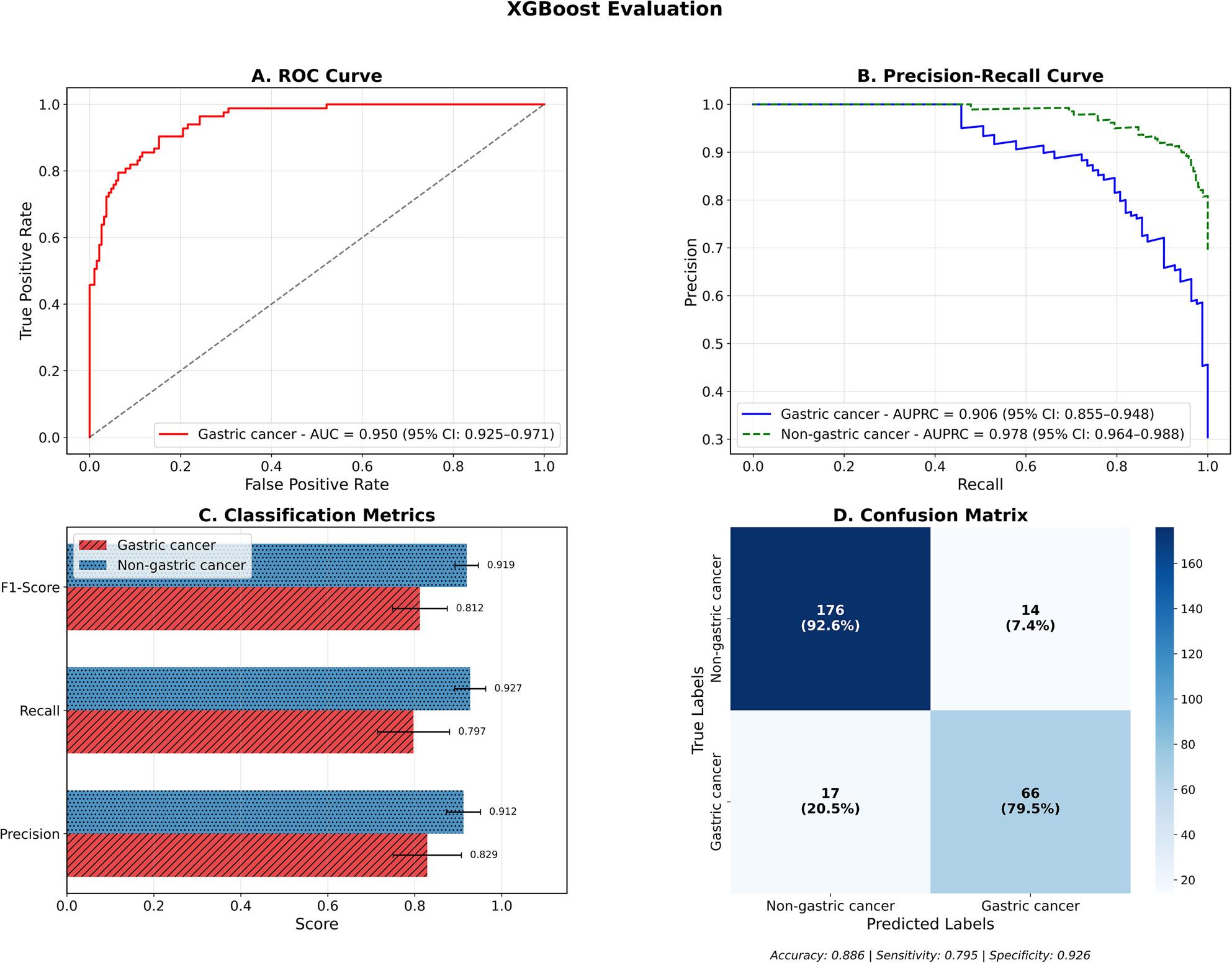
Performance evaluation of XGBoost model for gastric cancer vs. non-gastric cancer classification **A** ROC curve demonstrating superior discriminative performance with AUC of 0.950 (95% CI: 0.925–0.971). **B** Precision-recall curves showing excellent performance for both gastric cancer (AUPRC = 0.906, 95% CI: 0.855–0.948) and non-gastric cancer (AUPRC = 0.978, 95% CI: 0.964–0.988) classes. **C** Classification metrics revealing high F1-scores, recall, and precision across both classes. **D** Confusion matrix indicating improved classification accuracy of 88.6%, sensitivity of 79.5%, and specificity of 92.6%

Building on the baseline logistic regression model’s performance, we evaluated XGBoost to improve the detection of gastric cancer. XGBoost displayed superior performance over the baseline, with the confusion matrix revealing correct classification of 66 out of 83 gastric cancer cases (79.5% sensitivity) and 176 out of 190 non-gastric cancer cases (92.6% specificity). The model produced 17 false negatives (20.5% of gastric cancer cases misclassified as non-gastric) and 14 false positives (7.4% of non-gastric cancer cases misclassified as gastric), resulting in an overall accuracy of 0.886 (95% CI: 0.850–0.920) as shown in Fig. 3D. The model displayed excellent performance across key metrics, with precision values of 0.829 (95% CI: 0.750–0.907) and 0.912 (95% CI: 0.871–0.950) for gastric and non-gastric cancer classes respectively. The recall performance showed improved gastric cancer detection at 0.797 (95% CI: 0.711–0.877) and maintained high non-gastric cancer recall of 0.927 (95% CI: 0.889–0.960). This resulted in F1-scores of 0.812 (95% CI: 0.745–0.871) and 0.919 (95% CI: 0.891–0.945) for gastric and non-gastric cancer classes respectively (Fig. 3C). The model achieved strong AUPRC values of 0.906 (95% CI: 0.855–0.948) for gastric cancer and 0.978 (95% CI: 0.964–0.988) for non-gastric cancer classes (Fig. 3B). The discriminatory power denoted by an AUROC of 0.950 (95% CI: 0.925–0.971) exceeded the baseline performance (Fig. 3A).

#### Random forest

**Fig. 4:**
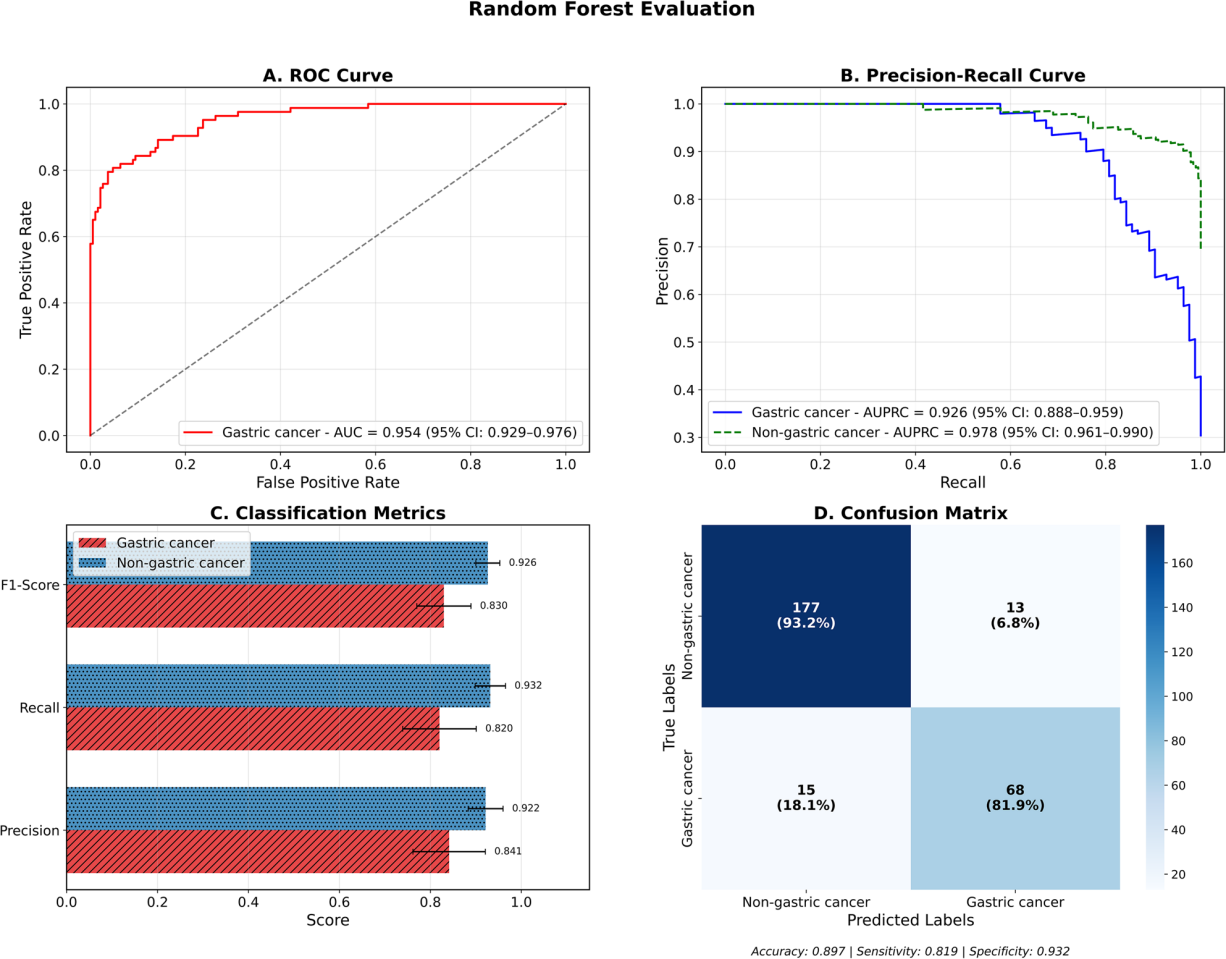
Performance evaluation of Random Forest model for gastric cancer vs non-gastric cancer classification **A** ROC curve showing excellent discriminative capacity with AUC of 0.954 (95% CI: 0.929-0.976). **B **Precision-recall curves demonstrating robust performance for gastric cancer (AUPRC = 0.926, 95% CI: 0.888-0.959) and non-gastric cancer (AUPRC = 0.978, 95% CI: 0.961-0.990) classification. **C** Classification performance metrics indicating balanced precision and recall across both classes. **D** Confusion matrix showing the highest overall accuracy of 89.7%, with sensitivity of 81.9% and specificity of 93.2%

Random Forest achieved the highest overall performance among all tested models, with the confusion matrix showing correct classification of 68 out of 83 gastric cancer cases (81.9% sensitivity) and 177 out of 190 non-gastric cancer cases (93.2% specificity). The model produced 15 false negatives (18.1% of gastric cancer cases misclassified as non-gastric) and 13 false positives (6.8% of non-gastric cancer cases misclassified as gastric), resulting in an overall accuracy of 0.897 (95% CI: 0.861–0.930) as shown in Fig. 4D. The model displayed excellent balanced performance across key metrics, with precision values of 0.841 (95% CI: 0.753–0.912) for gastric cancer and 0.922 (95% CI:0.881–0.957) for non-gastric cancer classification. The recall performance displayed the highest gastric cancer detection at 0.820 (95% CI: 0.734–0.896) and robust non-gastric cancer recall of 0.932 (95% CI: 0.897–0.963). This resulted in F1-scores of 0.830 (95% CI: 0.767–0.886) and 0.926 (95% CI: 0.898–0.951) for gastric and non-gastric cancer classes respectively (Fig. 4C). The model achieved excellent AUPRC values of 0.926 (95% CI: 0.888–0.959) for gastric cancer and 0.978 (95% CI: 0.961–0.990) for non-gastric cancer classes (Fig. 4B). The discriminatory power denoted by an AUROC of 0.954 (95% CI: 0.929–0.976) was the highest among all tested models (Fig. 4A). Detailed performance metrics with confidence intervals are provided in Supplementary Table S8 and the classification report is provided in Supplementary Table S9.

#### Model comparison and statistical validation

All the metrics for the models were calculated using the selected feature set (*n* = 93) of both demographic and genomic variables. Both black-box models outperformed the baseline, with Random Forest achieving superior performance in gastric cancer detection (sensitivity: 82.0% for Random Forest vs. 79.7% for XGBoost vs. 73.5% for baseline) and overall accuracy (89.7% vs. 88.6% vs. 81.3% respectively). Model calibration analysis revealed improved probability confidence for both black-box models, with XGBoost achieving a Brier score of 0.087 and ECE of 0.069 and Random Forest achieved Brier score of 0.078 and ECE of 0.062 after calibration, representing 51% and 56% improvements in Brier score over the baseline Brier score of 0.165 respectively (Supplementary Figures S1, S2 and S3). Both calibration metrics consistently demonstrated that Random Forest provided the most reliable probability estimates, with its ECE of 0.062 indicating that predicted probabilities deviated from true outcomes by only 6.2% points on average across probability bins. The superior performance of both black-box models, particularly Random Forest’s enhanced gastric cancer detection capability combined with its excellent calibration metrics, demonstrates the effectiveness of tree-based algorithms for this complex classification task.

DeLong’s test was performed to statistically evaluate the differences between the areas under two ROC curves derived from distinct models. The difference in the AUROC of XGBoost and the baseline logistic regression was statistically significant (*p* < 0.001, Z = -5.06). Similarly, the difference in AUROC of Random Forest and the baseline logistic regression was statistically significant (*p* < 0.001, Z = -4.94). Both ensemble models displayed statistically significant improvements over the baseline logistic regression model.

### Feature type-specific model evaluation

While statistical literature is widely available for controlling confounding factors through regressing-out analyses, it is not applied to predictive ensemble models due to the non-linear assumptions that they rely on [40]. The ShapRFECV-based feature selection step applied after appropriate preprocessing had identified 93 optimal features, of which 2 were demographic features (age, geographic location) with the rest being genomic features. Due to the inability to directly regress out confounding variables (like age and other demographic factors) for non-linear models like XGBoost and Random Forest, all the 3 machine learning models were trained and evaluated on three distinct feature sets:

- Demographic features only (age and geographic location).
- Genomic features only (excluding all demographic variables).
- Combined feature set (both demographic and genomic features).

Models that were trained exclusively on demographic features showed poor predictive performance with AUROC, AUPRC, and gastric cancer-specific recall values of the demographic-only logistic regression model being 0.657 (95% CI: 0.589–0.720), 0.453 (95% CI: 0.359–0.552), and 0.534 (95% CI: 0.423–0.646) respectively. The AUROC, AUPRC and and gastric cancer-specific recall values of the demographic-only Random Forest model were 0.735 (95% CI: 0.675–0.791), 0.468 (95% CI: 0.38–0.563) and 0.388 (95% CI: 0.282–0.493) respectively. The AUROC, AUPRC, and gastric cancer-specific recall values of the demographic-only XGBoost model were 0.783 (95% CI: 0.727–0.837), 0.573 (95% CI: 0.466–0.678), and 0.453 (95% CI: 0.333–0.544) respectively.

Models that were trained exclusively on genomic features showed better predictive performance with AUROC, AUPRC, and gastric cancer-specific recall values of the genomic-only logistic regression model being 0.802 (95% CI: 0.742–0.854), 0.628 (95% CI: 0.519–0.733), and 0.712 (95% CI: 0.614–0.808) respectively. The AUROC, AUPRC and and gastric cancer-specific recall values of the genomic-only Random Forest model were 0.947 (95% CI: 0.918–0.972), 0.919 (95% CI: 0.875–0.955) and 0.820 (95% CI: 0.734–0.896) respectively. The AUROC, AUPRC, and gastric cancer-specific recall values of the genomic-only XGBoost model were 0.938 (95% CI: 0.906–0.965), 0.900 (95% CI: 0.848–0.941), and 0.808 (95% CI: 0.721–0.883) respectively.

The above results indicate a 23.6% performance increase of AUROC values of the genomic-only models compared to the demographic-only models. This demonstrates that demographic features contribute minimally to the model performance compared to the genomic features, the primary drivers of model performance, which is also supported by the fact that 91 out of the 93 features selected by ShapRFECV were genomic features. Complete performance metrics including precision, recall are presented in Supplementary Table S10.

### Subgroup performance and sensitivity analyses

#### Geographic stratification analysis

To assess potential domain shift due to geographic imbalance in the dataset, model performance was evaluated separately across European, North American, South American, and Asian cohorts. The majority of samples in the test set (*n* = 273) originated from Europe (*n* = 169), followed by North America (*n* = 61), South America (*n* = 25), and Asia (*n* = 18). In the Asian cohort, all samples belonged to the non-gastric cancer group in the test set, preventing calculation of AUROC, AUPRC, sensitivity, or F1-score for that region.

For the logistic regression model, performance was highest in Europe (AUROC = 0.866, 95% CI: 0.803–0.923; AUPRC = 0.715, 95% CI: 0.584–0.837) and moderate in North America (AUROC = 0.742, 95% CI: 0.604–0.867; AUPRC = 0.589, 95% CI: 0.381–0.775). Performance declined in South America (AUROC = 0.542, 95% CI: 0.308–0.787; AUPRC = 0.643, 95% CI: 0.381–0.877), likely due to small sample size (*n* = 25) and higher variance. The Random Forest model performed the best for all three regions (Europe, North America, and South America), with the highest performance in Europe again (AUROC = 0.986, 95% CI: 0.969–0.997; AUPRC = 0.976, 95% CI: 0.949–0.994) and moderate in North America (AUROC = 0.781, 95% CI: 0.646–0.899; AUPRC = 0.661, 95% CI: 0.439–0.838). Performance was markedly better for South America (AUROC = 0.938, 95% CI: 0.826-1; AUPRC = 0.952, 95% CI: 0.862-1) likely due to gastric cancer and non-gastric cancer samples being balanced. Performance was intermediate for the XGBoost model, with Europe displaying the highest performance (AUROC = 0.98, 95% CI: 0.958–0.995; AUPRC = 0.965, 95% CI: 0.926–0.99), followed by South America (AUROC = 0.887, 95% CI: 0.731–0.994; AUPRC = 0.919, 95% CI: 0.783–0.995) and North America (AUROC = 0.813, 95% CI: 0.698–0.913; AUPRC = 0.582, 95% CI: 0.364–0.806).

Full stratified results for all models are provided in Supplementary Table S11.

#### Age-stratified calibration analysis

Age-stratified calibration analysis was performed to evaluate model reliability across patient subgroups. Patients were divided into three age groups based on gastric cancer epidemiology: < 45 years (*n* = 90; Gastric cancer = 20, Non-gastric cancer = 70; mean age: 34.6 ± 8.0), 45–65 years (*n* = 124; Gastric cancer = 35, Non-gastric cancer = 89; mean age: 53.7 ± 5.6), and ≥ 65 years (*n* = 59; Gastric cancer = 28, Non-gastric cancer = 31; mean age: 73.1 ± 5.4).

Calibration metrics varied in the youngest age group across all models (Logistic Regression: Brier = 0.130, ECE = 0.102; XGBoost after calibration: Brier = 0.055, ECE = 0.101; Random Forest after calibration: Brier = 0.053, ECE = 0.069). XGBoost showed excellent calibration before sigmoid correction (Brier = 0.046, ECE = 0.032), though calibration slightly degraded after sigmoid correction, suggesting the model was already well-calibrated for this subgroup. Random Forest achieved the best calibration; despite class imbalance (22% gastric cancer prevalence), all models produced reasonable probability estimates.

In the middle-aged cohort (45–65 years), there was optimal calibration across all models. Better calibration was achieved in ensemble models: XGBoost (Brier = 0.085, ECE = 0.048) and Random Forest (Brier = 0.082, ECE = 0.052). These are the lowest ECE values across all age ranges and could represent the most reliable probability estimate. The superior calibration is likely due to a more balanced class distribution (28% gastric cancer prevalence) and a larger sample size (*n* = 124). Logistic regression displayed moderate calibration (Brier = 0.178, ECE = 0.129), and performed less optimally than calibrated ensemble models in the same age range.

In the oldest age group (≥ 65 years), uncalibrated models showed erratic calibration patterns. Sigmoid calibration improved the calibration metrics for the XGBoost model (Brier = 0.142, ECE = 0.051), while Random Forest (Brier = 0.106, ECE = 0.086) achieved acceptable calibration metrics. While there is some residual irregularity in the calibration curves, they might reflect our limited sample size (*n* = 59) constraining stable probability estimation. The overall calibration metrics remain within acceptable ranges for ensemble models, while logistic regression showed the poorest calibration in this age group (Brier = 0.191, ECE = 0.156).

Across all age groups, sigmoid calibration improved ensemble probability estimates. The middle-aged group (45–65 years) has the most reliable calibration due to increased sample size and class balance. Complete age-stratified calibration curves for all three models are presented in Supplementary Figures S4-S6.

#### Batch effect evaluation and data source sensitivity analysis

Two complementary sensitivity analyses were conducted to evaluate whether potential batch effects from different data sources (NCBI vs. EnteroBase) affected model performance: source-stratified performance evaluation on the test set and leave-one-source-out (LOSO) cross-validation.

##### Principal component analysis of data sources

Principal Component Analysis (PCA) on the complete encoded feature set (*n* = 1,362 samples) was performed to visualize potential batch effects arising from different data sources. PCA showed substantial overlap between NCBI and EnteroBase samples in the reduced dimensional space, with the first two principal components (PC1 and PC2) explaining 15.6% and 5.9% of variance respectively (Supplementary Figure S7). The sample distributions from both sources were largely intermixed instead of forming distinct clusters, suggesting limited systematic technical variation between sources. Quantitative assessment using silhouette score, which measures cluster separation (values near 0 indicate overlapping distributions and values near 1 indicate distinct clusters), yielded a score of 0.008 for data source labels. This value, which is very close to zero, confirms that NCBI and EnteroBase samples are not systematically separated in the feature space. This indicates that batch effects from different sequencing platforms, assembly methods, or collection contexts do not dominate the genomic and demographic feature distributions, and that observed feature patterns indicate genuine biological variation and not source-specific technical artifacts.

##### Source-stratified performance on test set

When evaluated separately on NCBI-derived (*n* = 121) and EnteroBase-derived (*n* = 152) samples within the held-out test set, all three models showed robust performance across both sources. For Logistic Regression, the AUROC was 0.762 (95% CI: 0.666–0.849) for NCBI and 0.879 (95% CI: 0.819–0.934) for EnteroBase samples, with corresponding AUPRC values of 0.524 (95% CI: 0.382–0.719) and 0.784 (95% CI: 0.655–0.891), thus showing a consistent discriminatory ability. XGBoost achieved an AUROC of 0.896 (95% CI: 0.840–0.947) for NCBI and 0.978 (95% CI: 0.957–0.993) for EnteroBase, with an AUPRC of 0.772 (95% CI: 0.632–0.891) for NCBI and AUPRC of 0.963 (95% CI: 0.924–0.988) for EnteroBase. Random Forest showed an AUROC of 0.893 (95% CI: 0.824–0.956) for NCBI and 0.984 (95% CI: 0.968–0.995) for EnteroBase with the corresponding AUPRC values being 0.826 (95% CI: 0.711–0.921) and 0.973 (95% CI: 0.943–0.992). The consistency in performance metrics across the various data sources suggests that any potential batch effects do not significantly impair model predictions on the held-out test set.

##### Leave-One-Source-Out (LOSO) cross-validation

By training on one source and testing on the entirely held-out other source, LOSO analysis provided a more stringent evaluation of cross-source generalization. When trained on NCBI samples (*n* = 581) and tested on EnteroBase samples (*n* = 781), logistic regression achieved an AUROC of 0.757 (95% CI: 0.718–0.795) and an AUPRC value of 0.615 (95% CI: 0.550–0.681). The XGBoost model achieved an AUROC of 0.770 (95% CI: 0.724–0.811) and an AUPRC of 0.735 (95% CI: 0.683–0.783), and Random Forest achieved an AUROC of 0.802 (95% CI: 0.761–0.840) and an AUPRC of 0.762 (95% CI: 0.713–0.808). Conversely, when trained on EnteroBase samples and tested on NCBI samples, the performance still remained consistent for all the models: logistic regression (AUROC = 0.736 (95% CI: 0.696–0.775), AUPRC = 0.516 (95% CI: 0.448–0.587)), XGBoost (AUROC = 0.870 (95% CI: 0.833-0.900), AUPRC = 0.782 (95% CI: 0.713–0.838)), and Random Forest (AUROC = 0.879 (95% CI: 0.844–0.908), AUPRC = 0.825 (95% CI: 0.773–0.867)).

Complete source-stratified metrics and LOSO cross-validation results are provided in Supplementary Tables S12 and S13.

### Model explanations

The global SHAP summary plots provide post-hoc global explanations of the model to identify the features that contributed most to the model’s predictions.

**Fig. 5:**
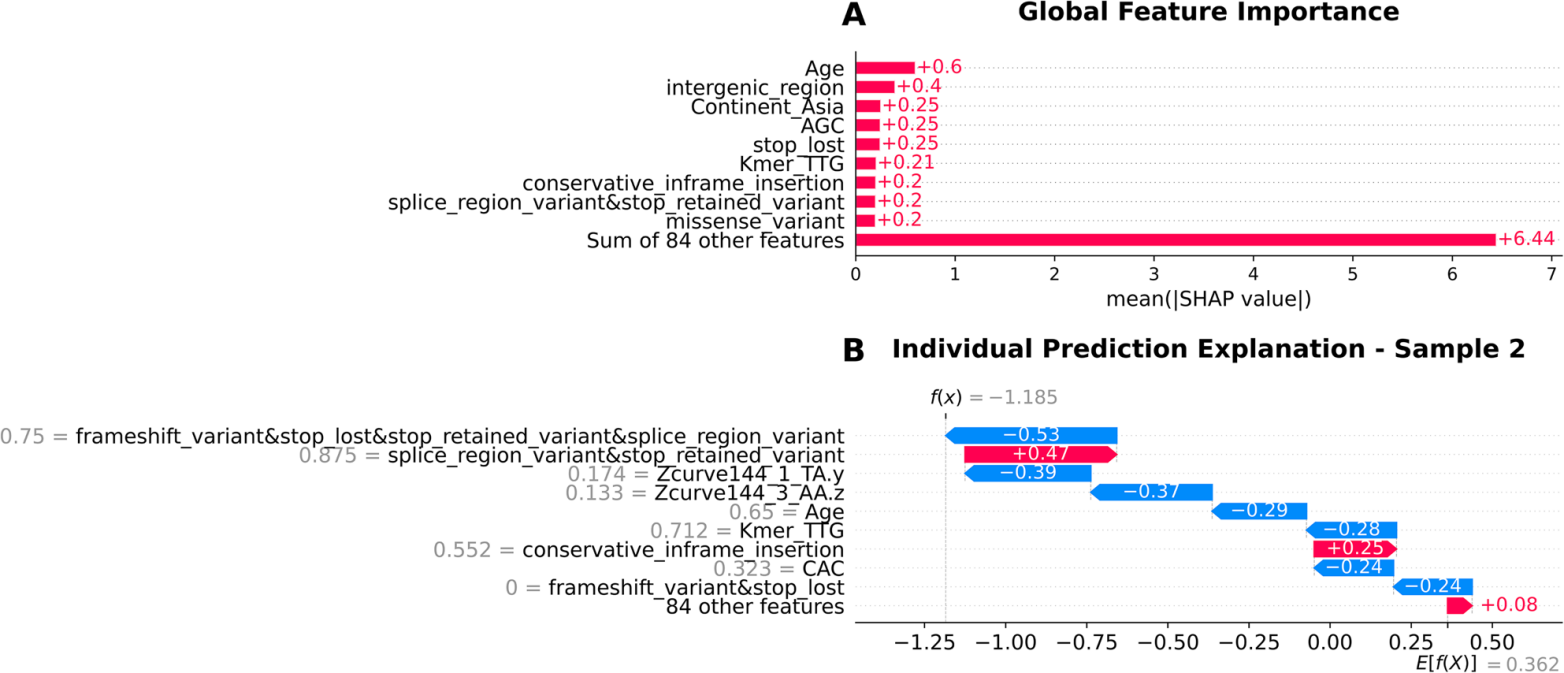
SHAP (SHapley Additive exPlanations) analysis of logistic regression model interpretability **A** Global feature importance showing the mean absolute SHAP values across all features, with Age (0.60) and Continent_Asia (0.25) as the most influential predictors, while 84 additional features collectively contribute 6.44 to model predictions. **B** Individual prediction explanation for Sample 2 (f(x) = -1.185, E[f(X)] = 0.362) demonstrating how specific feature values contribute toward non-gastric cancer prediction (red bars, positive values) or gastric cancer prediction (blue bars, negative values), with the overall negative prediction score indicating classification toward gastric cancer (class 0)

In the case of the baseline logistic regression model, the model primarily relied on demographic variables, with Age as the most influential predictor, followed by Continent Asia. Aggregate variant categories such as intergenic_region, conservative_inframe_insertion and other combinations of different variant categories also contributed, though with moderate impact (Fig. 5A). Sequence-derived features such as k-mers TTG and reverse k-mer AGC were contributing factors, while Z-curve was not in the top 10 influential features for the baseline model.

**Fig. 6:**
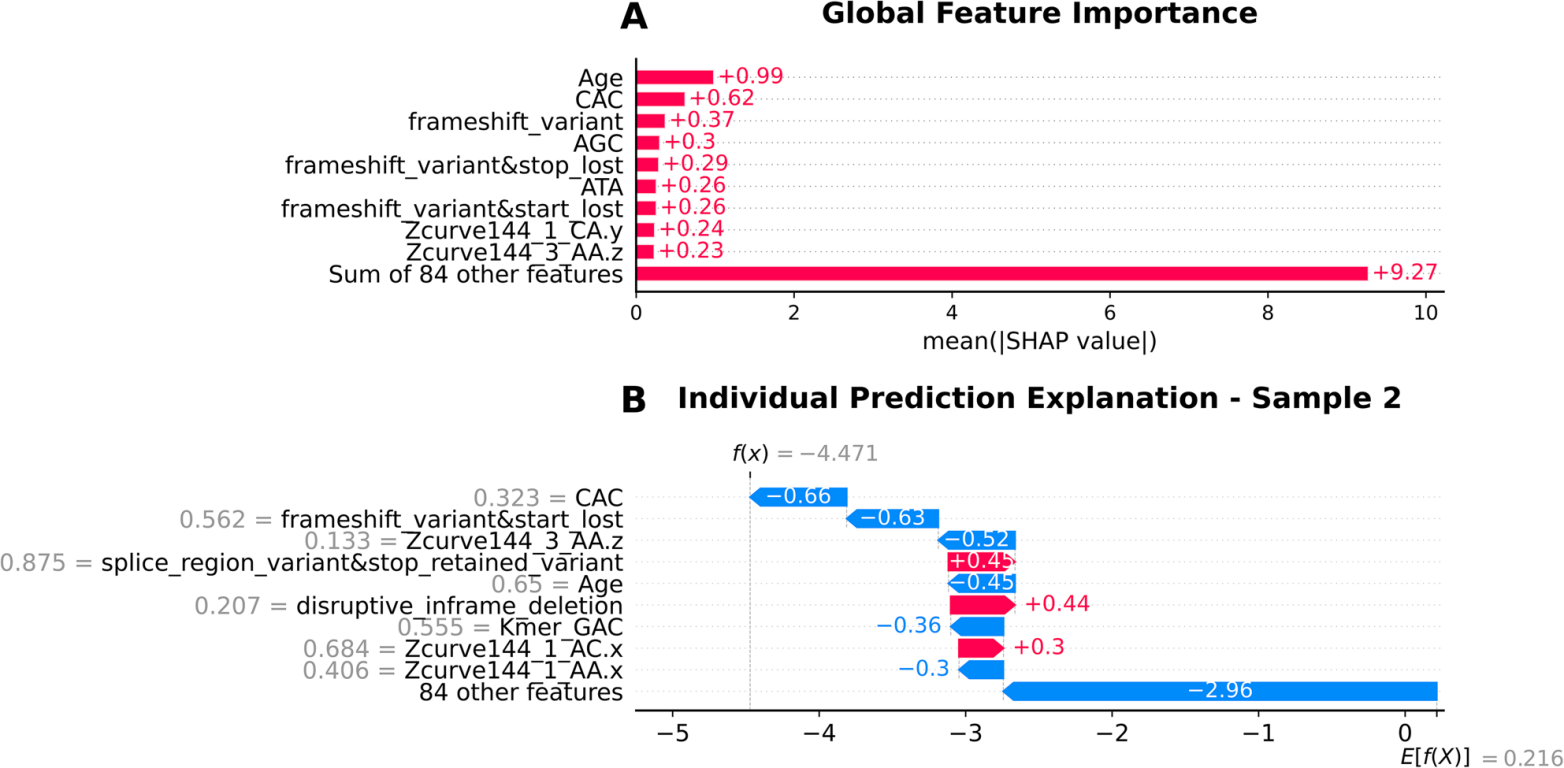
SHAP analysis revealing feature importance and prediction explanations for the XGBoost model **A** Global feature importance highlighting Age (0.99) as the dominant predictor, followed by CAC (0.62) and frameshift_variant (0.37), with 84 other features contributing 9.27 collectively. **B** Individual prediction explanation for Sample 2 (f(x) = -4.471, E[f(X)] = 0.216) showing how CAC (-0.66), frameshift variants (-0.63), and age-related features (-0.52) drive the prediction strongly toward gastric cancer (class 0), while some variant categories provide modest contributions toward non-gastric cancer classification

The XGBoost model captured a combination of demographic, aggregate variant categories and sequence-derived features. Age was the top predictor here too followed by a sequence-derived feature CAC k-mer. Among variant categories, frameshift_variant and frameshift_variant&stop_lost had considerable contributions, while other sequence k-mers such as AGC, ATA, and GTA also ranked highly. In addition to the above-mentioned features, Z-curve descriptors (Zcurve144_1_CA.y and Zcurve144_3_AA.z) appeared among the top 10 influential predictors, suggesting that genome composition features provided additional discriminatory power (Fig. 6A).

**Fig. 7:**
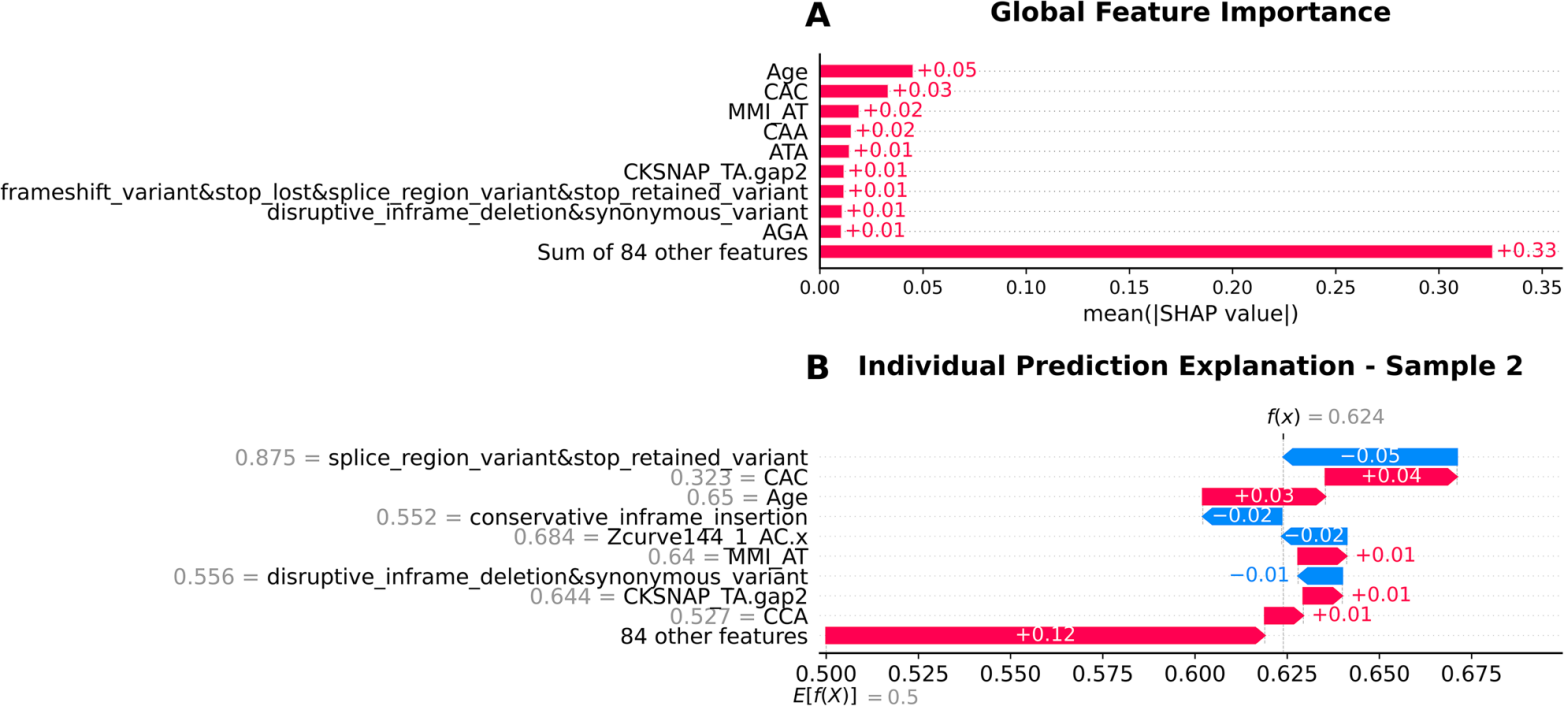
SHAP analysis demonstrating the interpretability of the Random Forest model predictions **A** Global feature importance showing relatively balanced feature contributions with Age (0.05), CAC (0.03), and MMI_AT (0.02) as leading predictors, and 84 other features contributing 0.33 collectively, indicating more distributed feature utilization compared to other models. **B** Individual prediction explanation for Sample 2 (f(x) = 0.624, E[f(X)] = 0.5) revealing subtle feature contributions, with splice_region_variant providing influence toward gastric cancer (-0.05) and CAC contributing toward non-gastric cancer (+0.04), with the overall positive prediction score indicating classification toward non-gastric cancer (class 1)

Similar to XGBoost, Age and CAC k-mer were the top predictors, indicating their consistent importance across models. The Random Forest also emphasized sequence descriptors, such as MMI_AT, CAA, and CKSNAP_TA.gap2, alongside variant categories like frameshift_variant&stop_lost&splice_region_variantstop_retained_variant and disruptive_inframe_deletion&synonymous_variant. Additional k-mers (ATA, AGA) and structural variant categories (for e.g., conservative_inframe_insertion) contributed, though with smaller SHAP values compared to Age and CAC (Fig. 7A).

Across all models, age consistently emerged as the most influential predictor, while Continent_Asia was particularly important in the logistic regression model. Tree-based classifiers (XGBoost and Random Forest) captured a broader range of sequence k-mers and variant categories, suggesting they were better able to integrate complex genomic patterns along with demographic features. The top 10 features of all three models are listed in Supplementary Table S14 with their mean |SHAP| values, mean SHAP values (indicating direction of effect), standard deviations, and median absolute SHAP values.

To interpret individual-level predictions, we generated SHAP waterfall plots for representative test samples that were classified as gastric cancer (positive class, encoded as 0) by each model. These plots illustrate how genomic and demographic features contributed positively or negatively to the probability of gastric cancer in correctly predicted cases.

The logistic regression model relied heavily on combined variant types, with stop_lost&disruptive_inframe_deletion&synonymous_variant, k-mers and age as primary features that contribute to gastric cancer classification. The 84 aggregated features contributed moderately, while splice_region_variant&stop_retained_variant provided the strongest opposing influence (Fig. 5B).

The XGBoost model emphasized sequence composition, with CAC and frameshift_variant&start_lost as top contributors toward gastric cancer prediction. Z-curve parameter, Zcurve144_3_AA.z, age, and k-mer GAC played a significant role. The 84 aggregated features showed strong gastric cancer contribution, while splice_region_variant&stop_retained_variant, disruptive_inframe_deletion, and Zcurve144_1_AC.x contributed to the non-gastric cancer class (Fig. 6B).

The Random Forest model showed variant categories pushing the classification towards the gastric cancer class with splice_region_variant&stop_retained_variant being a comparatively strong contributor, while the remaining variant categories such as conservative_inframe_insertion and disruptive_inframe_deletion&synonymous_variant show moderate contributions. Genome composition features like Z-curve descriptors (Zcurve144_1_AC.x) also contributed to the gastric cancer class (Fig. 7B).

#### Feature overlap analysis

A feature overlap analysis was performed to identify which features in the top 10 features overlap across the models, with SHAP being employed as a unified model-agnostic framework to carry out this analysis.

All three models independently identified Age as the most important feature, with substantially higher SHAP values than any other predictor (mean |SHAP|: 0.601 for logistic regression, 0.985 for XGBoost, 0.045 for Random Forest; Supplementary Figure S8). This fairly robust cross-model agreement strongly validates that Age is the dominant predictor of gastric cancer risk in our *H. pylori* genomic dataset, independent of model architecture or algorithmic approach.

Beyond Age, the models also showed partial consensus on important predictive features. Among the top 10 features, we observed: (i) 2 features shared between XGBoost and Random Forest (CAC k-mer, ATA k-mer), (ii) 1 feature shared between XGBoost and logistic regression (AGC k-mer), and (iii) 1 feature shared between Random Forest and logistic regression (conservative_inframe_insertion). Only Age appeared in the top 10 features across all three models (Supplementary Figure S8). The modest pairwise overlaps (20–30%) are indicative of fundamental differences in how linear and tree-based models capture predictive patterns, with ensemble models identifying important features through non-linear relationships and interactions.

To confirm the reliability of SHAP-based feature importance, we also compared SHAP values against traditional coefficient magnitudes for logistic regression. The Spearman correlation was highly significant (ρ = 0.915, *p* < 0.001; Supplementary Figure S9, left panel), which validates that SHAP can accurately capture feature importance in linear models. Cross-model SHAP comparisons revealed moderate correlations: XGBoost vs. Random Forest (ρ = 0.501, *p* < 0.001), XGBoost vs. logistic regression (ρ = 0.382, *p* < 0.001), and Random Forest vs. logistic regression (ρ = 0.271, *p* < 0.001; Supplementary Figure S9). These moderate correlations are expected given the architectural differences between linear and tree-based models.

Logistic regression emphasized features with strong linear marginal effects, including intergenic_region variants (mean |SHAP| = 0.396), geographic origin (Continent_Asia, mean |SHAP| = 0.253), and specific variant types (stop_lost, mean |SHAP| = 0.247). In contrast, ensemble models prioritized k-mer sequence patterns (CAC, ATA, AGC) and Zcurve144 that may reflect non-linear sequence signatures. For instance, the XGBoost model highlighted complex variant combinations (frameshift_variant&stop_lost, frameshift_variant&start_lost), which could suggest that the model hinges on interaction effects between multiple genomic alterations, while the Random Forest model identified several distinct features including MMI_AT and disruptive_inframe_deletion&synonymous_variant, which is indicative of the complementary nature of insights from the different model architectures (Supplementary Figure S8).

## Discussion

Three machine learning algorithms were used to identify primarily genomic variables that contributed to the development of gastric cancer in individuals infected with *H. pylori*. In order to be inclusive and accessible to clinicians, white-box models were used in addition to high-performance black-box machine learning models. A variety of explainability measures like SHAP were also utilized to explain identified associations and increase clinician confidence, and this framework was designed to uncover dynamic genomic risk factors and supplement clinician decision-making. This is demonstrably superior to traditional modeling methods which are constrained by the number of variables that can be incorporated into the study and unable to unearth complex associations.

It can be seen that age was consistently identified as the top predictor of gastric cancer across all three models. This aligns with existing scientific literature implicating older patients as more susceptible to developing gastric cancer, with around 6 out of every 10 people diagnosed with gastric cancer being 65 years or older [32]. Calibration quality, however, varied across different age strata. In the most clinically important cohort of middle-aged patients (45–65 years, *n* = 124), where early detection has immense benefits due to increased gastric cancer incidence, the ensemble models showed excellent calibration after sigmoid correction (ECE = 0.048–0.052 for ensemble models). The younger age group (< 45 years, *n* = 90), although accounting for fewer than 10% of gastric cancer cases and thus a lower clinical priority, also displayed good calibration after correction (ECE ≤ 0.101). In elderly patients (≥ 65 years, *n* = 59), calibration metrics remained within acceptable ranges (ECE ≤ 0.086 for Random Forest), although the smaller sample size resulted in less smooth calibration curves, which is a common challenge in predictive modeling for limited subgroup sample sizes.

The top 3 predictors in the baseline model were age, belonging to Asia and silent mutations in the start codons (Fig. 5A). Fatality linked to gastric cancer has been shown to be particularly high in regions of East Asia and Eastern Europe [41]. The malignancy of certain geographical strains could be in part due to differences in virulence genotypes like *cagA* EPIYA motifs [42]. The silent mutations in the start codon could lead to the production of proteins with altered functional properties, thus influencing the malignancy of the *H. pylori* strain.

The top 3 contributing factors in the XGBoost model were age, the reverse kmer CAC and the simultaneous presence of frameshift mutations and loss of stop codons (Fig. 6A). The presence of the trinucleotide CAC could be indicative of distinctive pathogenicity islands or similar genomic signatures associated with *H. pylori* infection and adaptation. For example, pathogenicity islands in *H. pylori* exhibit atypical nucleotide composition and codon usage compared to the core genome [43]. This could explain why a particular k-mer like CAC was flagged as an important feature, and could potentially mark a horizontally acquired virulence region, and thus marked differences between strains. Cancer-associated *H. pylori* strains might lack or gain certain AT-rich regions, which modify the frequency of a GC-heavy k-mer like CAC. While we cannot pinpoint a single gene or motif conferring virulence, the k-mers could be interpreted as reflective of general sequence composition. Such k-mer based signals have been used in previous Genome Wide Association Studies to identify pathogenic variants [44], and are fairly indicative of broader genomic differences between strains.

Stop codons have also been implicated in existing literature, with a study depicting the loss of expression of the virulence gene *babA* due to a mutation resulting in a stop codon being acquired [45]. The loss of stop codons could be likely to capture phase variation events that control the expression of key infection factors and thus influencing gastric cancer risk.

The top 3 contributing factors in the Random Forest model were age, the reverse k-mer CAC and the presence of AT rich regions (Fig. 7A). While the first two features were also the most important in the XGBoost model, multivariate mutual information aids in capturing higher-order nucleotide correlations beyond regular k-mer counts. The high correlation with AT could indicate the difference in the degree of sequence-order through structured repeats and conserved motifs between cancer-associated and benign strains. This is biologically plausible since certain virulence-associated loci of *H. pylori* display unusual AT content, and this abundance of AT-rich sequences may signal the presence of pathogenic genomic segments which distinguish more virulent strains [43].

The two black-box models also consistently identified the presence of the CAC, ATA and AAA reverse k-mers as associated with increased discriminatory power between infection outcomes. As short DNA motifs, these k-mers might be proxies for larger genomic differences between the strains. Both the k-mers ATA and AAA are indicative of AT-rich content characteristic of some genomic islands in *H. pylori*, while the enrichment of the CAC k-mer could indicate codon usage bias or the prevalence of repetitive sequence motifs found in high-virulence lineages.

Combinations of variants have also been marked as gastric-cancer inducing factors in both the XGBoost and Random Forest models, such as the high presence of all 4 mutations- frameshift variants, loss of stop codons, missense variants and splice region variants. These variants result in an alteration of amino acids in bacterial proteins, and could reflect divergent strains, altered virulence factors and genetic plasticity. Previous studies have found that many common allelic variants are more frequent in outer-membrane associated virulence genes, like *sabA* and *babA* [46]. Since a single point mutation was found to be capable of preventing BabA expression during infection, similar such mutations in genes could be selected for in certain hosts, thereby contributing to gastric cancer. This is suggestive of the fact that cancer-associated strains can harbor disruptive mutations in certain genes that could alter proteins, and thus prove beneficial for pathogenicity in the gastric niche. *H. pylori* is also known to regulate virulence genes via phase variation through frame-shifting insertions and deletions. The *oipA* gene contains a CT dinucleotide repeat in its signal peptide region, and strain-to-strain differences in the number of repeats cause frameshifts that dictate whether the *oipA* gene is ‘off’ or ‘on’. Additionally, this ‘on’ *oipA* in-frame status also occurs along with *cagA* in more virulent strains [47, 48]. The models could be picking up phase differences in the cancer-derived strains. While they have limited links to known virulence mechanisms, recent studies have identified multiple SNPs and variants associated with gastric cancer isolates in DNA metabolism and outer membrane protein genes [44, 49]. The models picking up on other genomic descriptors like Z-curve descriptors and k-mer derivatives suggest that genomic architecture and lineage contribute to risk. Different *H. pylori* lineages like hpEastAsia and hpEurope have differences in oligonucleotides, distinct alleles of virulence genes and evolutionary histories [50, 51]. One study found that *H. pylori* strains from gastric cancer patients were 3 to 11 times more likely to possess a 7-gene integrative conjugative element [52], which would in turn influence the Z-curves. The models might be detecting the genomic fingerprints of such mobile elements associated with high risk.

Besides corroborating existing literature on the causative risk factors of malignant *H. pylori* infection induced gastric cancer and thus establishing itself as reliable, our study can also advance research that seeks to detect and mitigate *H. pylori* outcomes through personalized treatment approaches. Further studies that seek to investigate causal inference between identified features can also make use of our explainable machine learning framework. Since the primary focus in the clinical setting hinges around lifestyle factors, we believe additionally accounting for pathogen genomics through whole genome sequencing of patient *H. pylori* samples could complement existing efforts to predict a patient’s risk of the infection progressing to gastric cancer.

Methodologically, our use of both interpretable (white-box) and high-performance (black-box) models with SHAP explanations follow recent recommendations for clinical AI. There is a growing consensus that explainability is essential in healthcare settings; explainable AI techniques have been shown to increase clinician confidence and meet regulatory transparency requirements by revealing the reason behind a model’s prediction [53]. For example, SHAP, which was employed by us, is a widely used post-hoc method that attributes scores to individual features and helps in making the model’s reasoning more transparent [38]. Our framework, which combines ensemble ML with explanatory plots, is reflective of the TRIPOD + AI guidelines which integrates open, interpretable methods, even within complex models [54].

Although the models possessed remarkable discriminatory power, a variety of pertinent features were not incorporated into the model. It has been shown that family history, smoking habits, diet and other clinical features play a very important role in the development of *H. pylori* infection associated outcomes, along with the role of host immune factors [55]. One of the limitations of this study is geographic bias, since the data had a large number of European samples (870) which may affect the generalizability of the results by not accurately capturing lineage-specific genomic signatures critical for deploying the model in high-risk Asian populations. Since the data was publicly available, the other limitations include possible varying biases introduced at different stages of data collection including participation bias and potential batch effects. Although detailed sequencing metadata was not available for all the genomes, we attempted to address batch effect concerns through 3 approaches - PCA which showed a substantial overlap between NCBI and EnteroBase samples (silhouette score = 0.008), performance evaluation by source stratification which also showed consistent metrics across the different sources, and leave-one-source-out cross-validation which showed optimal discriminatory abilities when the models trained on one source were tested on another. Thus, while there is technical variation between sources, we believe our models are generalizable across different sequencing and data deposition contexts. Access to more diverse data sources, particularly from the Global South, which can be further integrated into the model could mitigate existing geographic bias and externally validate the model in diverse populations. However, the need for whole genome sequencing limits current clinical applicability of the model as risk factors like smoking and family history can be more easily assessed. At present, high-risk countries like those in East-Asia rely on non-genomic methods like the ‘ABC’ method based on *H. pylori* antibodies and serum pepsinogen levels to determine high-risk individuals requiring endoscopy [56]. We therefore propose this model as an additive tool in high-risk and research settings. As the models identify genomic markers that cannot be captured by virulence gene-specific PCR along with the lack of host factor information, genomic risk profiling can be combined with routine care. Integrated genomic diagnostics for *H. pylori* are rapidly developing, as evidenced by the next-generation sequencing method developed by Gillet et al. that successfully retrieved the *H. pylori* virulome and resistome directly from patient gastric tissue [57]. In the future, antibiotic resistance and genomic risk could be determined for a patient undergoing endoscopy for dyspepsia. In addition to guiding therapy for the patient, our model applied to such sequence data could be used to predict the patient’s cancer risk. Clinical deployment thus requires validation in geographically diverse cohorts, integration of host and environmental risk factors, as well as more diversified outcome evaluation that can differentiate between gastric cancer subtypes. Given the guidelines that recommend the eradication of *H. pylori* in all infected patients, a genomic model, when implemented in appropriate conditions, could assist in identifying patients at especially high risk that could benefit from heightened surveillance and intervention.

## Conclusions

Integrating machine learning with bedside treatment will be a significant stride in early detection and personalized diagnostic methods for identifying the severity of *H. pylori* infection responses. In an attempt to improve clinical trust in black-box models, explainable AI was used to break down top features to improve transparency. Among the evaluated models, the two black-box models achieved greater recall in the prediction of gastric cancer compared to the baseline logistic regression model. The top predictor was consistently found to be patient age across all three models, while different genomic markers were identified by each of the models. Incorporating genomic features of the host along with clinical metadata could enhance the predictive power and the robustness of the models, since current *H. pylori* prediction models focus largely on clinical features. As gene expression plays a major role in the development of infection, integration of transcriptomic data and host gut metagenomic data could account for gene expression and interaction of *H. pylori* with other gut microbes. The sequence-derived genomic features implicated in this study open avenues for further research into the *H. pylori* genome beyond the already implicated virulence genes to assess possible causation behind the associations found in this study. Assimilating real-time data gathering options for the model through collaboration with medical centers incorporating whole genome sequence analyses of *H. pylori*, including clinical feedback loops and refining the user interface can render it a powerful tool for spontaneous risk-assessment and bolster regional health infrastructure. The high accuracy of our research model in discriminating between severe and benign *H. pylori* infection outcomes underscore its potential impact in deploying it in resource-limited populations that are disproportionately plagued by *H. pylori* infections after carrying out necessary external validation.

## Supplementary Information

Supplementary Material 1:Supplementary Table S1: Clinical metadata of the patients. Includes patient demographics like their age, sex, their geographical location and the disease phenotype. Supplementary Table S2: Presence/absence data of H. pylori virulence genes implicated in gastric cancer. Supplementary Table S3: Sequence-derived features extracted from H. pylori genomes using iFeatureOmega and MathFeature. Supplementary Table S4: Aggregate variant categories of H. pylori genomes. Supplementary Table S5: The final all feature-types integrated dataset. Supplementary Table S6: ShapRFECV feature elimination report. Contains information about the selected and eliminated features based on the recall metric. Supplementary Table S7: Optimal hyperparameters selected using Bayesian optimization for XGBoost and Random Forest models. Supplementary Table S8: Detailed evaluation metrics for all three models with class-specific precision, recall, F1-score, AUROC and AUPRC scores with lower and upper confidence intervals. Supplementary Table S9: Classification reports of all three models. Supplementary Table S10: Detailed evaluation metrics (AUROC, AUPRC, Precision, Recall, F1) for all models trained (i) only on demographic features (ii) only on genomic features. Supplementary Table S11: Detailed evaluation metrics (AUROC, AUPRC, Precision, Recall, F1) across Logistic Regression, XGBoost and Random Forest stratified by geographical region. Supplementary Table S12: Model performance metrics stratified by data source on held-out test set. Supplementary Table S13: Leave-one-source-out (LOSO) cross-validation performance metrics. Supplementary Table S14: Quantitative SHAP summary tables that rank features by mean absolute SHAP values for all three models (Logistic Regression, XGBoost, and Random Forest). Each table presents the top 10 features along with their mean |SHAP| values, mean SHAP values (indicating direction of effect), standard deviations, and median absolute SHAP values.

Supplementary Material 2. Fig S1. Calibration plot of logistic regression classifier.

Supplementary Material 3. Fig S2. Calibration plots of extreme gradient boosting (XGBoost) classifier before and after calibration.

Supplementary Material 4. Fig. S3. Calibration plots of random forest classifier before and after calibration.

Supplementary Material 5. Fig. S4. Age-stratified calibration curves for Logistic Regression model.

Supplementary Material 6. Fig. S5. Age-stratified calibration curves for XGBoost model.

Supplementary Material 7. Fig. S6. Age-stratified calibration curves for Random Forest model.

Supplementary Material 8. Fig S7. Principal Component Analysis (PCA) of features by data source.

Supplementary Material 9. Fig S8. SHAP-based feature importance comparison across models.

Supplementary Material 10. Fig S9. Cross-method validation of SHAP-based feature importance.

## Data Availability

The *H. pylori* genomes analyzed in this study are publicly available on the National Center for Biotechnology Information (NCBI) GenBank repository (https://www.ncbi.nlm.nih.gov/genbank/) and EnteroBase (https://enterobase.warwick.ac.uk/). Accession numbers for all 1,363 genomes used in analyses are provided in Supplementary Table 1. The custom code and analysis pipelines used in this study are available on GitHub (https:/github.com/Venkatesh-99/HP_ML) and archived in Zenodo at (https:/doi.org/10.5281/zenodo.17085927).

## Appendix

3 $$\:Precision\:=\:\frac{TP}{TP\:+\:FP}$$

4 $$\:Recall\:\left(Sensitivity\right)\:=\frac{TP}{TP\:+\:FN}\:$$

5 $$\:F1\:=2\:\times\:\frac{Precision\:\times\:\:Recall}{Precision\:+\:Recall}\:$$

Where:

- *TP* = True Positives.
- *FP* = False Positives.
- *FN* = False Negatives.

## Abbreviations

H. pylori: Helicobacter pylori
XGBoost: eXtreme Gradient Boosting
SMOTE-NC: Synthetic Minority Over-sampling Technique for Nominal and Continuous
SHAP: SHapley Additive exPlanations
AUROC: Area Under Receiver Operating Characteristic curve
MALT: Mucosa-associated lymphoid tissue
WHO: World Health Organisation
cagA: cytotoxin-associated gene A
cagPAI: cag pathogenicity island
vacA: vacuolating cytotoxin A
BabA: blood group antigen binding adhesin
oipA: outer inflammatory protein A
sLe^X^: sialylated Lewis antigens
SRA: Sequence Read Archive
NAC: Nucleic Acid Composition
MMI: Multivariate Mutual Information
ROC: Receiver Operating Characteristic
AUC: Area Under the Curve
AUPRC: Area Under the Precision-Recall curve
AP: Average precision
