# Supplementary figures and images for: Predicting clinical outcomes in Helicobacter pylori-positive patients using supervised learning through the integration of demographic and genomic features
Source: BMC Gastroenterol. 2026 Jan 29;26:143. doi: 10.1186/s12876-025-04595-3 (PMC12922380; doi:10.1186/s12876-025-04595-3)

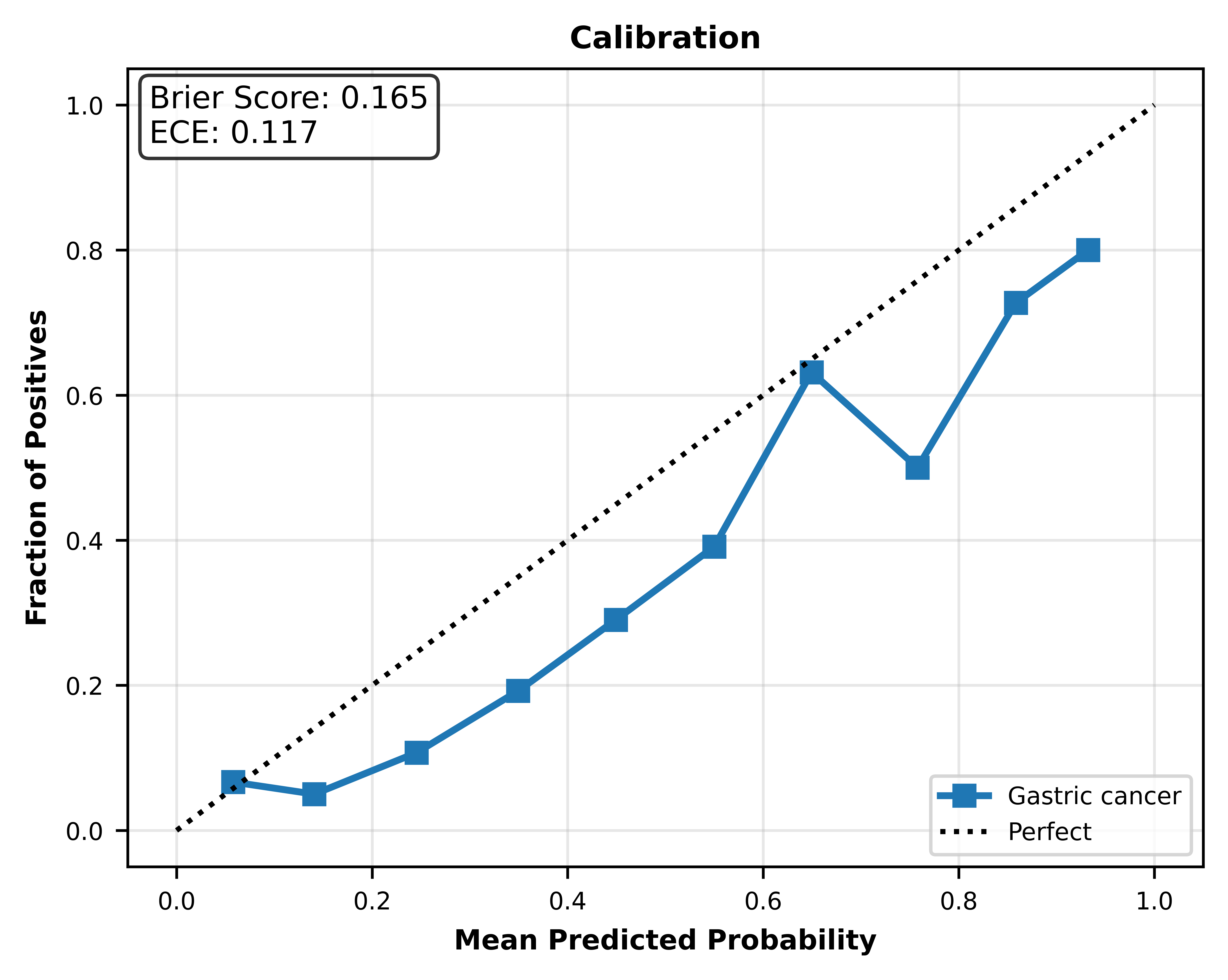

Supplement: Supplementary file 2 — Supplementary Material 2. Fig S1. Calibration plot of logistic regression classifier. [file 12876_2025_4595_MOESM2_ESM.png]

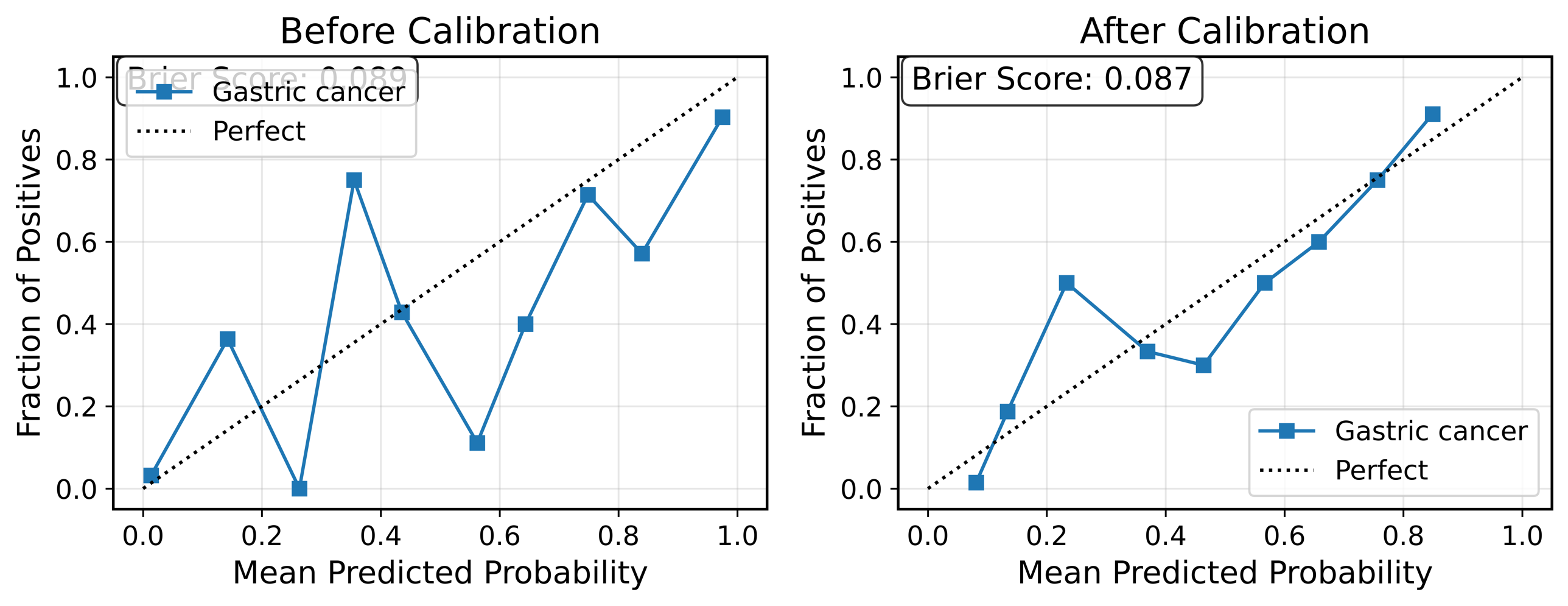

Supplement: Supplementary file 3 — Supplementary Material 3. Fig S2. Calibration plots of extreme gradient boosting (XGBoost) classifier before and after calibration. [file 12876_2025_4595_MOESM3_ESM.png]

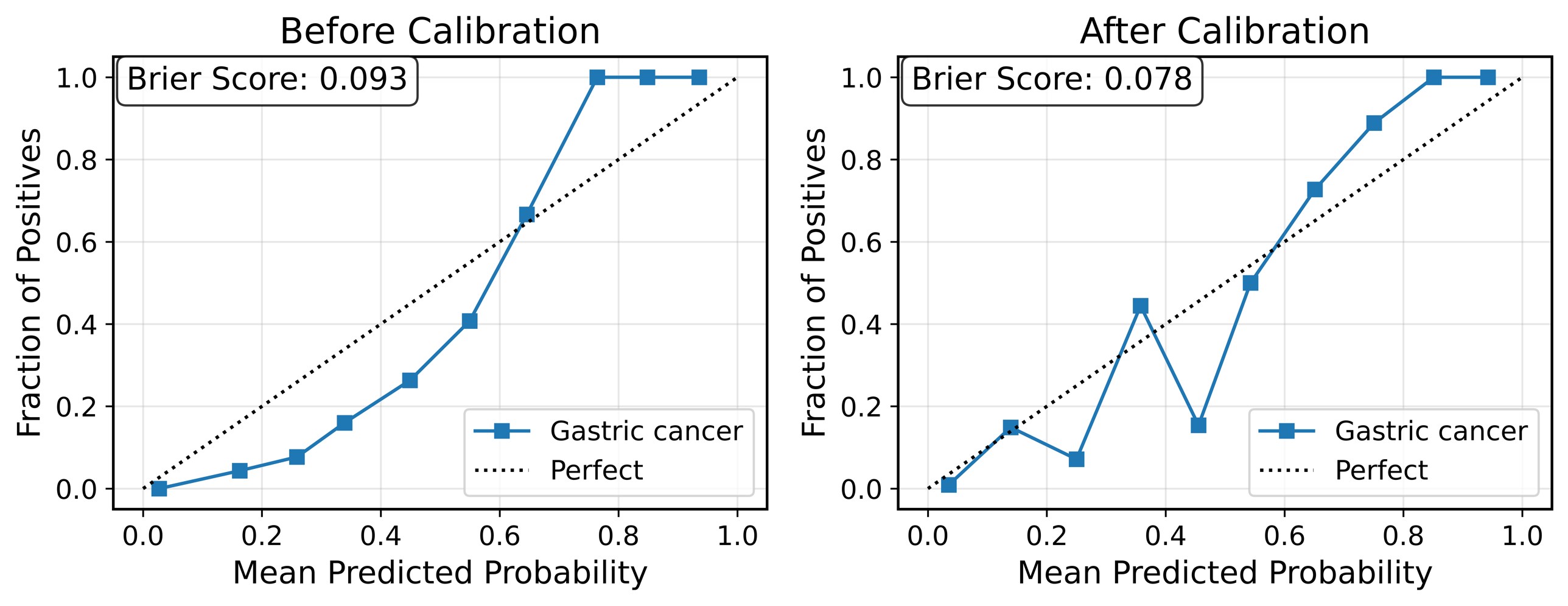

Supplement: Supplementary file 4 — Supplementary Material 4. Fig. S3. Calibration plots of random forest classifier before and after calibration. [file 12876_2025_4595_MOESM4_ESM.png]

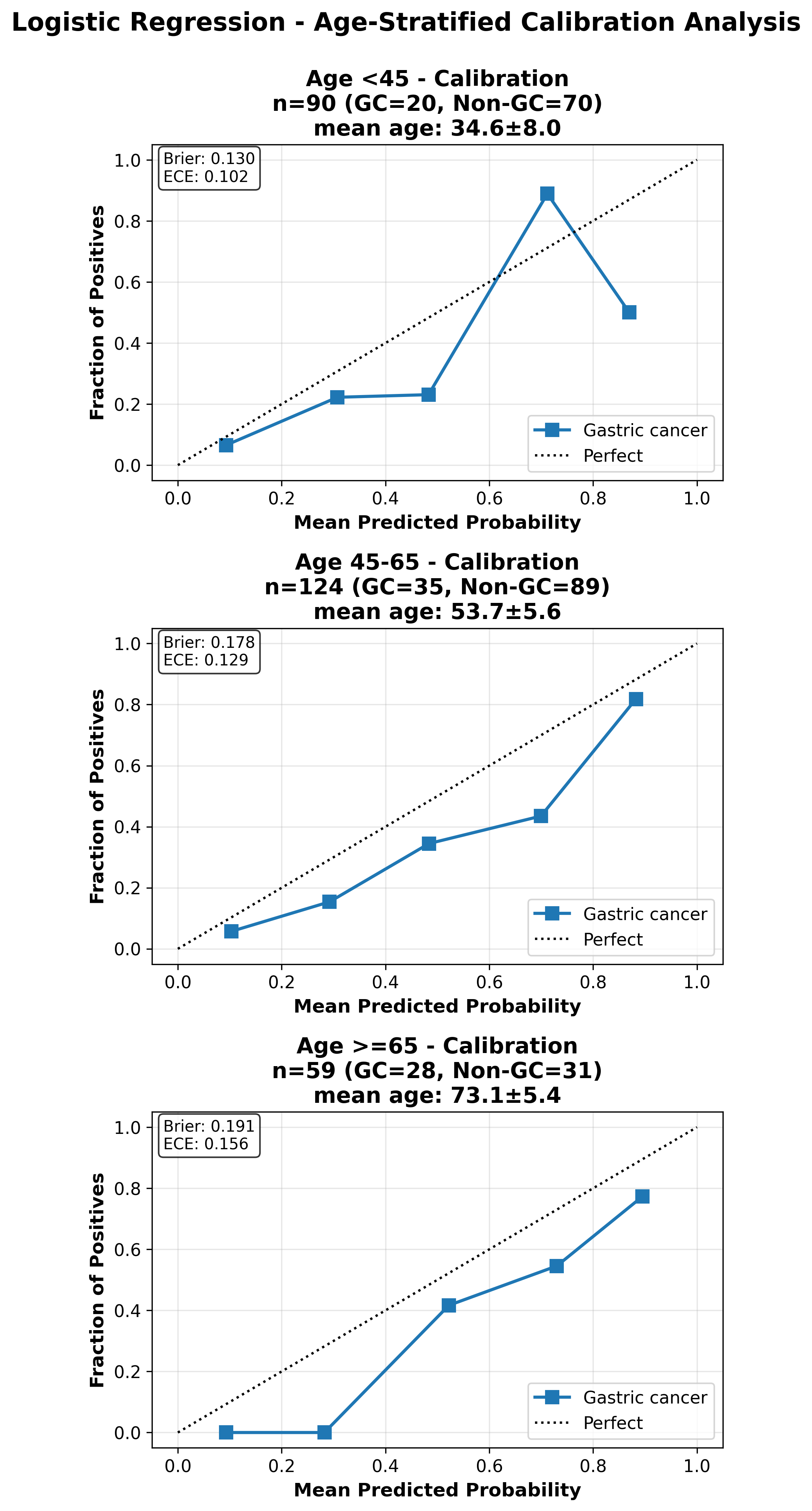

Supplement: Supplementary file 5 — Supplementary Material 5. Fig. S4. Age-stratified calibration curves for Logistic Regression model. [file 12876_2025_4595_MOESM5_ESM.png]

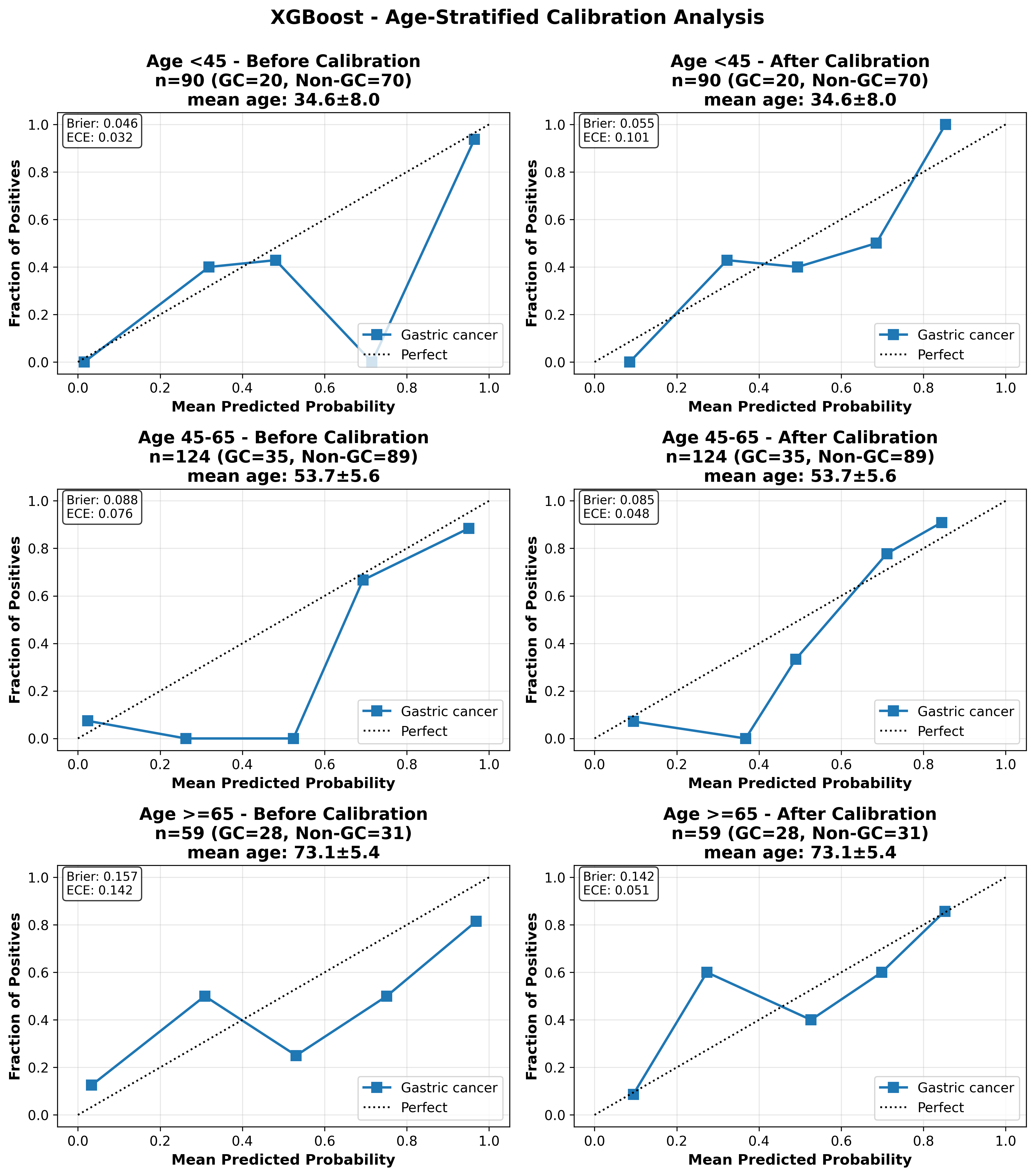

Supplement: Supplementary file 6 — Supplementary Material 6. Fig. S5. Age-stratified calibration curves for XGBoost model. [file 12876_2025_4595_MOESM6_ESM.png]

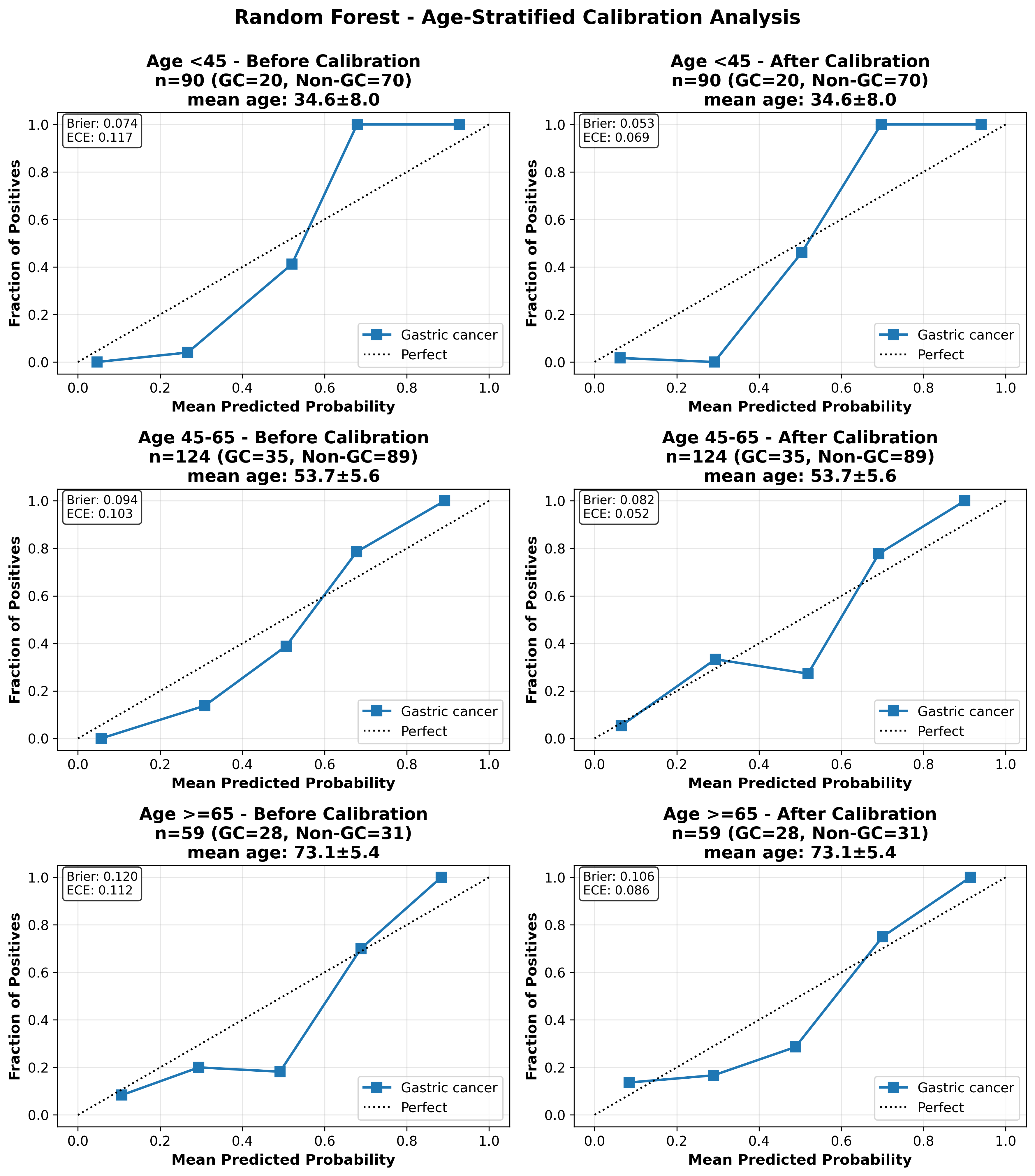

Supplement: Supplementary file 7 — Supplementary Material 7. Fig. S6. Age-stratified calibration curves for Random Forest model. [file 12876_2025_4595_MOESM7_ESM.png]

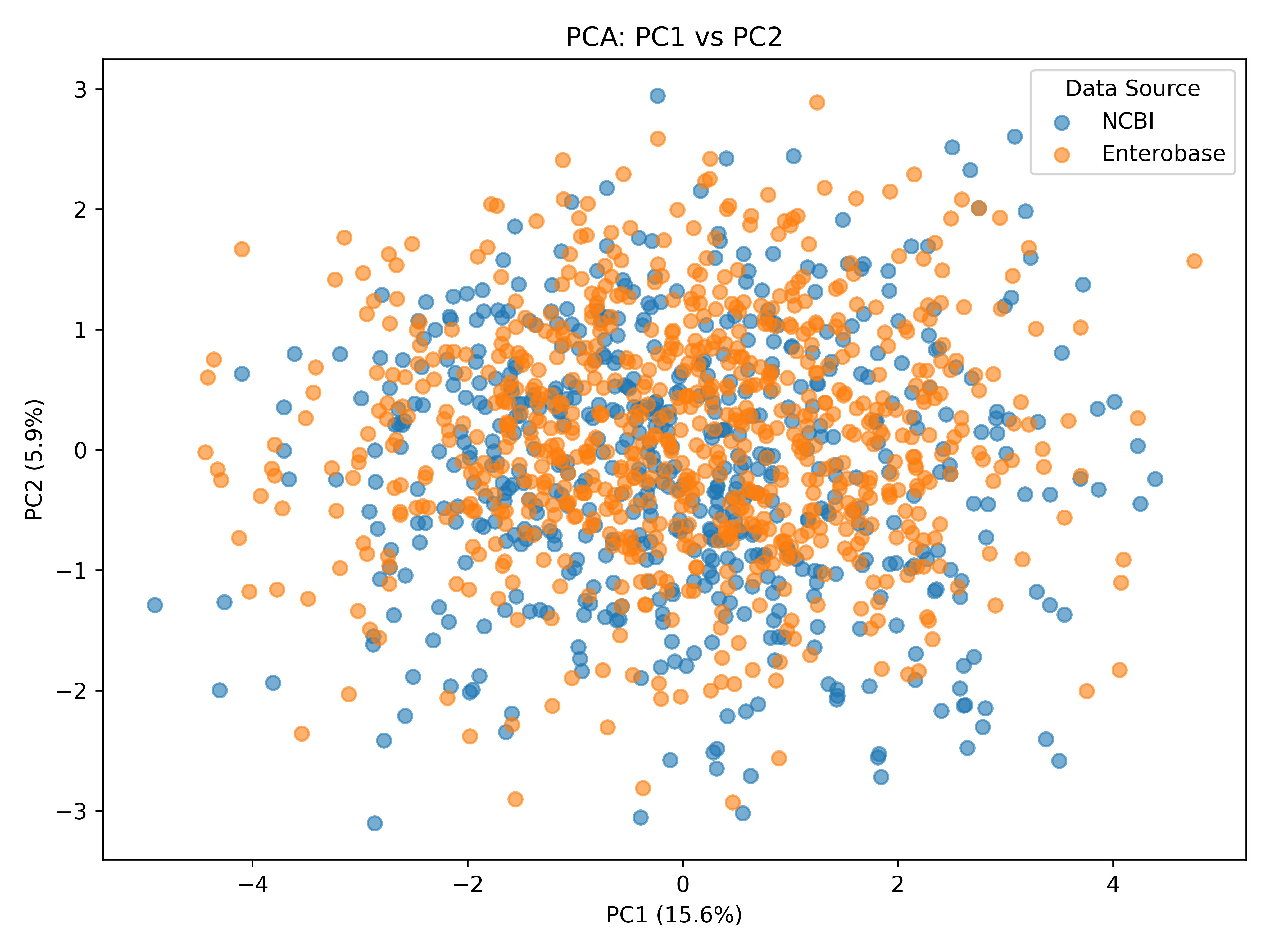

Supplement: Supplementary file 8 — Supplementary Material 8. Fig S7. Principal Component Analysis (PCA) of features by data source. [file 12876_2025_4595_MOESM8_ESM.png]

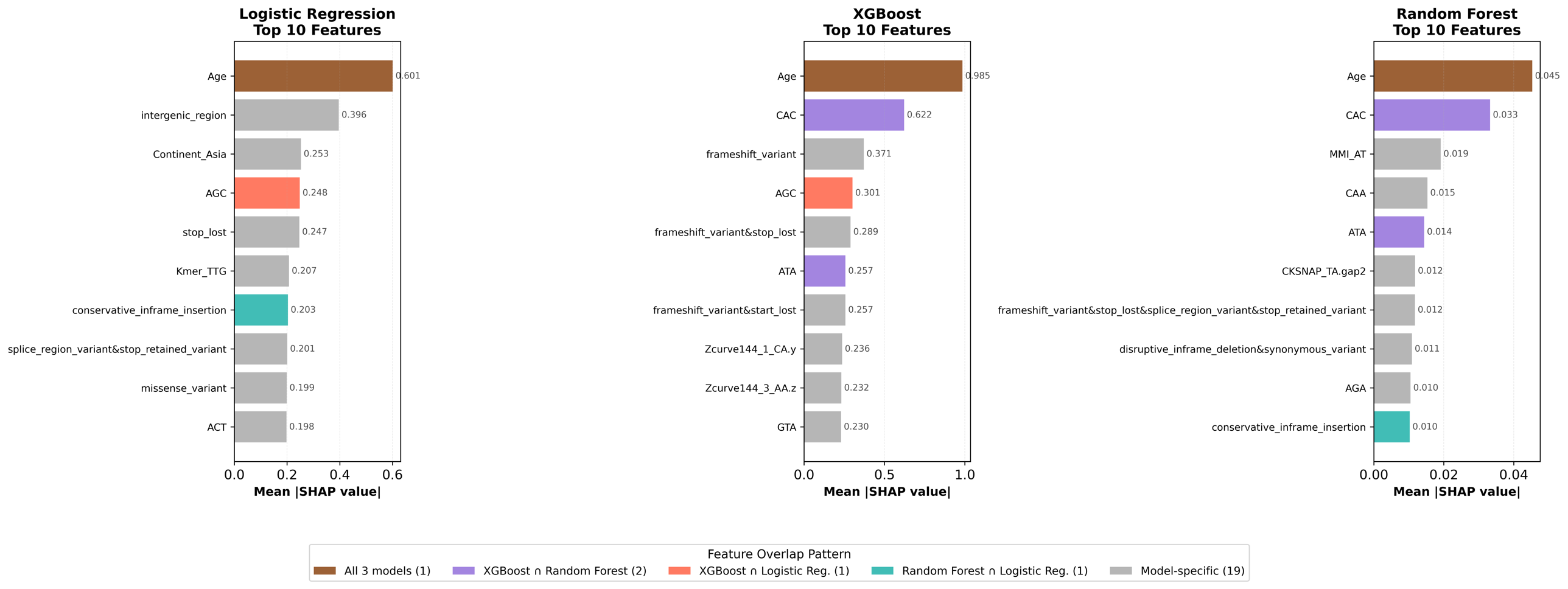

Supplement: Supplementary file 9 — Supplementary Material 9. Fig S8. SHAP-based feature importance comparison across models. [file 12876_2025_4595_MOESM9_ESM.png]

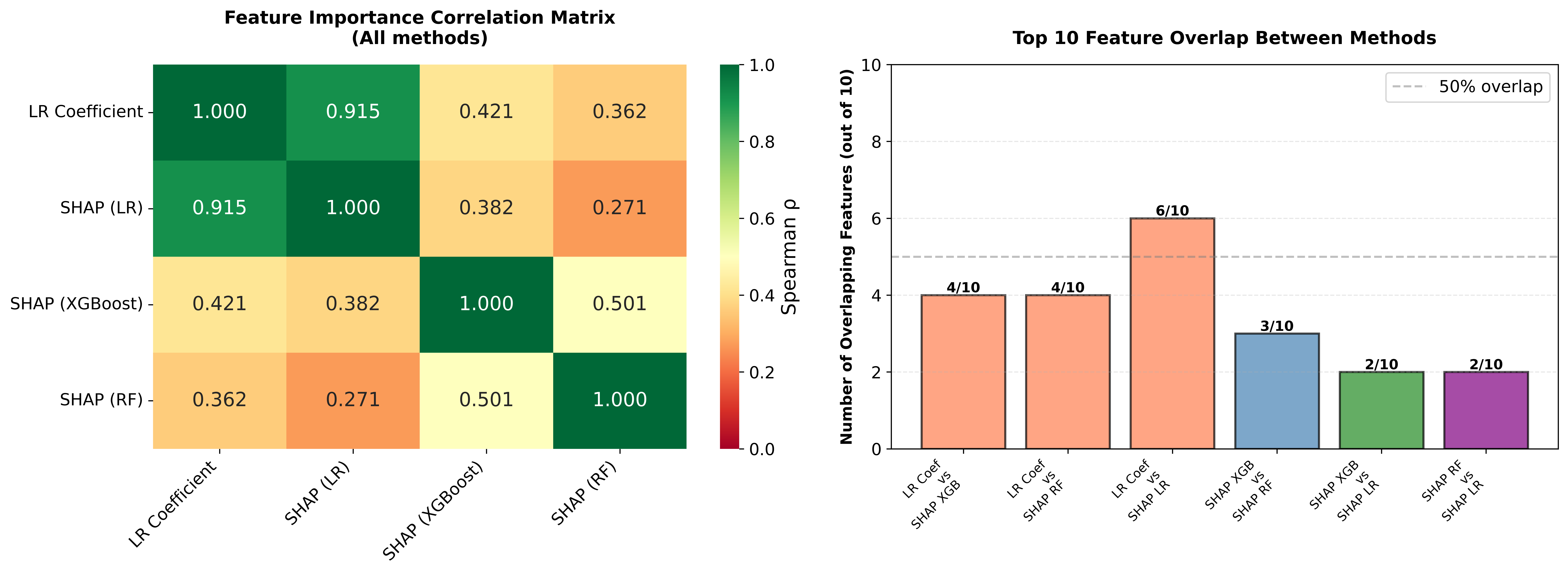

Supplement: Supplementary file 10 — Supplementary Material 10. Fig S9. Cross-method validation of SHAP-based feature importance. [file 12876_2025_4595_MOESM10_ESM.png]
